# Fast ripples reflect increased excitability that primes epileptiform spikes

**DOI:** 10.1093/braincomms/fcad242

**Published:** 2023-09-08

**Authors:** Shennan A Weiss, Itzhak Fried, Jerome Engel, Michael R Sperling, Robert K S Wong, Yuval Nir, Richard J Staba

**Affiliations:** Department of Neurology, State University of New York Downstate, Brooklyn, NY 11203, USA; Department of Physiology and Pharmacology, State University of New York Downstate, Brooklyn, NY 11203, USA; Department of Neurology, New York City Health + Hospitals/Kings County, Brooklyn, NY 11203, USA; Department of Neurosurgery, David Geffen School of Medicine at UCLA, Los Angeles, CA 90095, USA; Department of Neurosurgery, David Geffen School of Medicine at UCLA, Los Angeles, CA 90095, USA; Department of Neurology, David Geffen School of Medicine at UCLA, Los Angeles, CA 90095, USA; Department of Neurobiology, David Geffen School of Medicine at UCLA, Los Angeles, CA 90095, USA; Department of Psychiatry and Biobehavioral Sciences, David Geffen School of Medicine at UCLA, Los Angeles, CA 90095, USA; Brain Research Institute, David Geffen School of Medicine at UCLA, Los Angeles, CA 90095, USA; Departments of Neurology and Neuroscience, Thomas Jefferson University, Philadelphia, PA 19107, USA; Department of Physiology and Pharmacology, State University of New York Downstate, Brooklyn, NY 11203, USA; Department of Physiology and Pharmacology, Sackler School of Medicine, Tel Aviv University, Tel Aviv 6997801, Israel; Sagol School of Neuroscience, Tel Aviv University, Tel Aviv 6997801, Israel; Department of Biomedical Engineering, Faculty of Engineering, Tel Aviv University, Tel Aviv 6997801, Israel; The Sieratzki-Sagol Center for Sleep Medicine, Tel Aviv Sourasky Medical Center, Tel Aviv 6423906, Israel; Department of Neurology, David Geffen School of Medicine at UCLA, Los Angeles, CA 90095, USA

**Keywords:** high-frequency oscillation, fast ripple, ripple, epileptiform spike, neuron

## Abstract

The neuronal circuit disturbances that drive inter-ictal and ictal epileptiform discharges remain elusive. Using a combination of extra-operative macro-electrode and micro-electrode inter-ictal recordings in six pre-surgical patients during non-rapid eye movement sleep, we found that, exclusively in the seizure onset zone, fast ripples (200–600 Hz), but not ripples (80–200 Hz), frequently occur <300 ms before an inter-ictal intra-cranial EEG spike with a probability exceeding chance (bootstrapping, *P* < 1e−5). Such fast ripple events are associated with higher spectral power (*P* < 1e−10) and correlated with more vigorous neuronal firing than solitary fast ripple (generalized linear mixed-effects model, *P* < 1e−9). During the intra-cranial EEG spike that follows a fast ripple, action potential firing is lower than during an intra-cranial EEG spike alone (generalized linear mixed-effects model, *P* < 0.05), reflecting an inhibitory restraint of intra-cranial EEG spike initiation. In contrast, ripples do not appear to prime epileptiform spikes. We next investigated the clinical significance of pre-spike fast ripple in a separate cohort of 23 patients implanted with stereo EEG electrodes, who underwent resections. In non-rapid eye movement sleep recordings, sites containing a high proportion of fast ripple preceding intra-cranial EEG spikes correlate with brain areas where seizures begin more than solitary fast ripple (*P* < 1e−5). Despite this correlation, removal of these sites does not guarantee seizure freedom. These results are consistent with the hypothesis that fast ripple preceding EEG spikes reflect an increase in local excitability that primes EEG spike discharges preferentially in the seizure onset zone and that epileptogenic brain regions are necessary, but not sufficient, for initiating inter-ictal epileptiform discharges.

## Teaser

Weiss *et al*. report that fast ripples recorded in the intra-cranial EEG from epileptogenic regions can prime the initiation of after-going epileptiform spikes when associated with excessive neuronal firing. However, not all epileptogenic regions initiate epileptiform spikes this way.

## Graphical Abstract

**Graphical Abstract:**
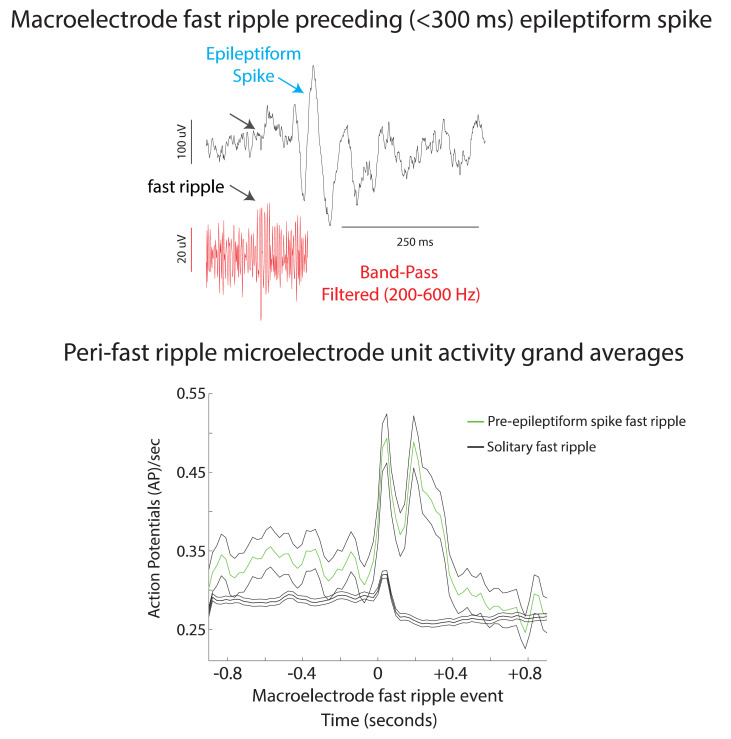

## Introduction

Spontaneous inter-ictal epileptiform spikes (i.e. spikes) were first recognized nearly a century ago in extracellular recordings from animals exposed to chemoconvulsants and in the EEG of patients with epilepsy.^1^ Spikes are of primary importance in the diagnosis of epilepsy,^2^ but also disrupt cognition,^3^ and may, in some cases, promote the development of spontaneous seizures.^4–6^ Epileptiform spikes, and their underlying neuronal correlates, are not uniform.^5–7^ Moreover, spikes propagate as travelling waves.^8,9^ Recent data suggest that epileptogenic brain regions may be identified by characterizing the spike initiation zone (SIZ) using precise source localization algorithms in intra-cranial EEG (iEEG).^9^ Little is known about what distinguishes spikes in the SIZ and what neuronal or macroscale neuronal network activity may prime an epileptiform discharge to occur next there.

High-frequency oscillations (HFOs) also are considered biomarkers of epileptogenic brain.^10^ HFOs are subcategorized as fast ripples (FRs, 200–600 Hz) and ripples (80–200 Hz). Like spikes FR, recorded by macroelectrodes, do not generally occur in healthy brain tissue,^11^ but unlike spikes FR are thought to better predict the development of chronic seizures in chemoconvulsant exposed animals^12^ and better identify the epileptogenic zone (EZ), which is necessary and sufficient for seizure generation in patients undergoing evaluation for epilepsy surgery.^13^ The physiological counterpart to FR is ripples that play a role in normal brain function, and specifically the sharp wave ripple complex in area CA1 of the hippocampus has been found to be essential for memory consolidation during non-REM (rapid eye movement) sleep.^14^ However, in chemoconvulsant animal models and in patients with epilepsy, ripples are generated at increased rates in and around the EZ.^15^ HFOs can occur superimposed on the background EEG [i.e. FR or ripple on oscillation (fRonO or RonO)] or superimposed on an inter-ictal epileptiform spike [i.e. FR or ripple on spike (fRonS or RonS)],^16,17^ and both are associated with ictogenesis in animal models^18,19^ and *in vitro*^6^ and *in vivo*^20^ recordings from epilepsy patients. However, fRonS and RonS may have increased accuracy for predicting epileptogenesis and localizing epileptogenic regions.^17,21–24^ Recently, we reported that fRonO can also spatially propagate in epileptogenic regions within a range <30 mm at a velocity of ∼1.54 mm/ms,^25,26^ and interestingly, propagation increased the probability that a fRonO was immediately followed by a spike. These data suggest excitability associated with fRonO could help generate spikes, but the neuronal activity contributing to fRonO and ensuing spike has not been studied.

**Figure 1:**
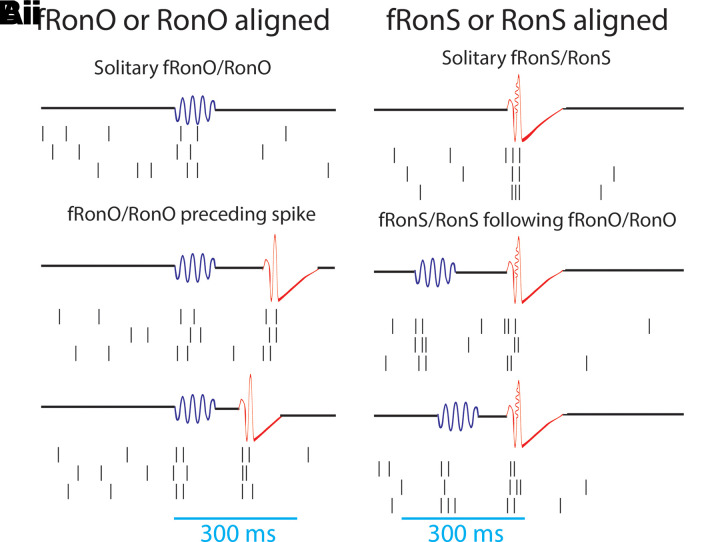
**Schematic of the analytical methods comparing unit APs with fRonO (**A**, blue), RonO (**A**, blue), fRonS (**B**, red) and RonS (**B**, red), as well as the temporal coincidence of these HFO event types within 300 ms (Aii, Bii).** AP trains were either temporally aligned to the onset for fRonO/RonO events (**A**) or the onset of fRonS/RonS events (**B**). Events were considered solitary if a fRonO or RonO did not precede a fRonS/RonS by <300 ms, and sharp spikes, lacking an HFO, were also accounted for (Aii). HFO event-unit trials aligned to fRonO/RonO events served to assess changes in excitability between solitary fRonO/RonO (Ai) and the fRonO/RonO that preceded (<300 ms) spikes (Aii). In contrast, HFO event-unit trials aligned to fRonS/RonS (**B**) assessed the changes in excitability between solitary fRonS/RonS (Bi) and fRonS/RonS that followed (<300 ms) fRonO/RonO (Bii).

To better understand and possibly establish a link between fRonO events and after-going spike probability, we investigated the neuronal and network activity associated with these events in patients with medically refractory focal epilepsy undergoing pre-surgical evaluation with paired macroelectrode and microelectrode implants. We compared neuronal action potentials (APs) with the properties of HFOs and corresponding local iEEG spikes, as well as HFO-spike temporal coincidence (Fig. 1). Our results show that in the SIZ, fRonO reflects increased neuronal excitability that precedes and promotes the generation of spikes restrained by inhibition. The putative SIZ almost always overlapped with epileptogenic regions, but not all epileptogenic regions were part of the SIZ as seizures often persisted after SIZ resection. These results bridge our understanding of clinically important inter-ictal biomarkers of epilepsy, offer important new insights to the origins of pathological brain activity and suggest that resecting the SIZ alone will not successfully control seizures.

## Materials and methods

### Dataset collection

All data were acquired with approval from the local institutional review board (IRB) at University of California Los Angeles (UCLA) and Thomas Jefferson University (TJU). The paired macroelectrode–microelectrode recording cohort was recorded from six patients with focal epilepsy at UCLA, who were implanted for the purpose of localization of the seizure onset zone (SOZ) in 2009–10.^27,28^ Sleep studies were conducted in the epilepsy monitoring unit 48–72 h after surgery and lasted 7 h on average, and sleep–wake stages were scored according to the established guidelines. The montage included two electrooculogram (EOG) electrodes; two EMG electrodes scalp electrodes at C3, C4 Pz and Fz; two earlobe electrodes used for reference; and continuous video monitoring. In each patient, 8–12 depth electrodes were implanted targeting medial brain areas. Both scalp and depth inter-ictal iEEG data, from the most medial depth electrode macroelectrode contact, were continuously recorded, during slow wave sleep, with a Stellate amplifier at a sampling rate of 2 kHz, bandpass-filtered between 0.1 and 500 Hz and re-referenced offline to the mean signal recorded from the earlobes. Each electrode terminated in eight 40 μm platinum–iridium microwires from which extracellular signals were continuously recorded (referenced locally to a ninth non-insulated microwire) at a sampling rate of 28 kHz using a Neuralynx Cheetah amplifier and bandpass-filtered between 1 and 6000 Hz.

The retrospective resection cohort used consecutive recordings selected from eight patients who underwent intra-cranial monitoring with depth electrodes between 2014 and 2018 at UCLA and from 15 patients at the TJU in 2016–18 for the purpose of localization of the SOZ.^29^ Inclusion criteria for this cohort included pre-surgical MRI for MRI-guided stereotactic electrode implantation, as well as a post-implant CT scan to localize the electrodes and stereo EEG (SEEG) recordings during non-REM sleep at a 2 kHz sampling rate and a post-resection/ablation MRI. Patients with no adequate post-operative clinical follow-up or a failure to record at least 10min of artefact-free iEEG during non-REM sleep were excluded. Eligible patients were identified through queries of pre-existing clinical databases. Post-implantation CT scans were co-registered and normalized with the pre-implant and post-resection MRIs using Advanced Neuroimaging Tools (https://picsl.upenn.edu/software/ants/) with neuroradiologist supervision, using an in-house pipeline.^30,31^ The position of each electrode contact was localized to normalized Montreal Neurological Institute (MNI) co-ordinates. For each patient, in the resection cohort clinical inter-ictal iEEG (0.1–600 Hz; 2000 samples per second) was recorded from 8 to 16 depth electrodes, each with 7–15 contacts, using a Nihon-Kohden 256-channel JE-120 long-term monitoring system (Nihon-Kohden America, Foothill Ranch, CA, USA) during epochs containing mostly high amplitude slow and delta oscillations. A larger number of electrodes with more contacts were implanted at TJU. The reference signal used for the recordings performed at UCLA was a scalp electrode position at Fz in the International 10–20 System. The reference signal used for the TJU recordings was an electrode in white matter.

### Spike sorting and characterization of HFOs and epileptiform spikes

APs were detected by high-pass filtering the local field potential (LFP) recordings above 300 Hz and applying a threshold at 5 SD above the median noise level. Detected events were further categorized as noise, single-unit, or multi-unit events using superparamagnetic clustering.^32^ Unit stability throughout sleep recordings was confirmed by verifying that spike waveforms, and inter-spike interval distributions were consistent and distinct throughout the night.

HFOs and sharp spikes were detected in the non-REM sleep iEEG using previously published methods^22,33,34^ (https://github.com/shenweiss). HFOs in the microwire LFP recording were not subject to analysis, minimizing the chance that high-frequency events such as FRs may be influenced by leakage from AP events.^35^ A two-stage algorithm first used a custom Hilbert-based detector implemented in Matlab with artefact rejection features to identify transient elevations in ripple (80–200 Hz) and FR (200–600 Hz) amplitude.^33^ In the second stage, the different HFO types: (i) ripples on oscillations (RonO), (ii) RonS, (iii) fRonO, (iv) fRonS and (v) sharp spikes without a HFO were distinguished using the topographical analysis of the wavelet convolution.^22,34,36^ In brief, this method identifies contours of power in the wavelet time–frequency transforms. Contours corresponding to power values less than a threshold defined by 0.2 × (max_time–frequency_-power − min_time–frequency_-power) + min_time–frequency_-power were removed. Each of the remaining contours was subsequently classified as a closed loop contour (CLC) if the contour’s first and last vertex co-ordinates were identical and an open loop contour if the first and last vertex were distinct. HFOs on spikes were distinguished by comparing onset of the outermost closed-loop isopower contour of the HFO in the time–frequency spectrogram with onset of the outermost open-loop isopower contour of the spike and determining if the latter occurred earlier. Open-loop contours without CLCs were designated sharp spikes without HFOs. This method also identified the spectral content, power and duration of each HFO. Ripples and FRs were distinguished by the mean frequency of the closed-loop contour group, and peak spectral content and power from the largest isopower contour value in the closed-loop contour group. Duration was computed from the onset and offset of the outermost contour in the closed-loop contour group. Compared with visual analysis of two reviewers, HFO and spike detection sensitivity and accuracy of event detection, using this method, was typically 80–90%.^33,34^ Following automatic detection of HFO and sharp spikes, false detections of clear muscle and mechanical artefact were deleted by visual review in Micromed Brainquick. This methodology did not detect bluntly contoured spikes, with no corresponding power signature >80 Hz, and sharp waves. In this study, we also did not sub-categorize HFOs on oscillations by oscillation subtype.^26,37^

To calculate the latency, within individual iEEG channels, between HFOs on oscillations and after-going epileptiform spikes, the onset times of all spikes, or a subtype of spikes, were subtracted from the onset time of each individual HFO on oscillation to find the minimum latency greater than zero. To determine if the distribution of these latencies exceeded chance, the number of HFO on oscillations were kept constant, but the onset times of the HFOs on oscillations across the recording time were uniformly randomized, within artefact free epochs, and resampled 500 times within each individual iEEG channel and across all iEEG channels. The HFOs were randomly assigned to any time and not restricted to occur with oscillations.

### Preparing GLMMs

**Figure 2:**
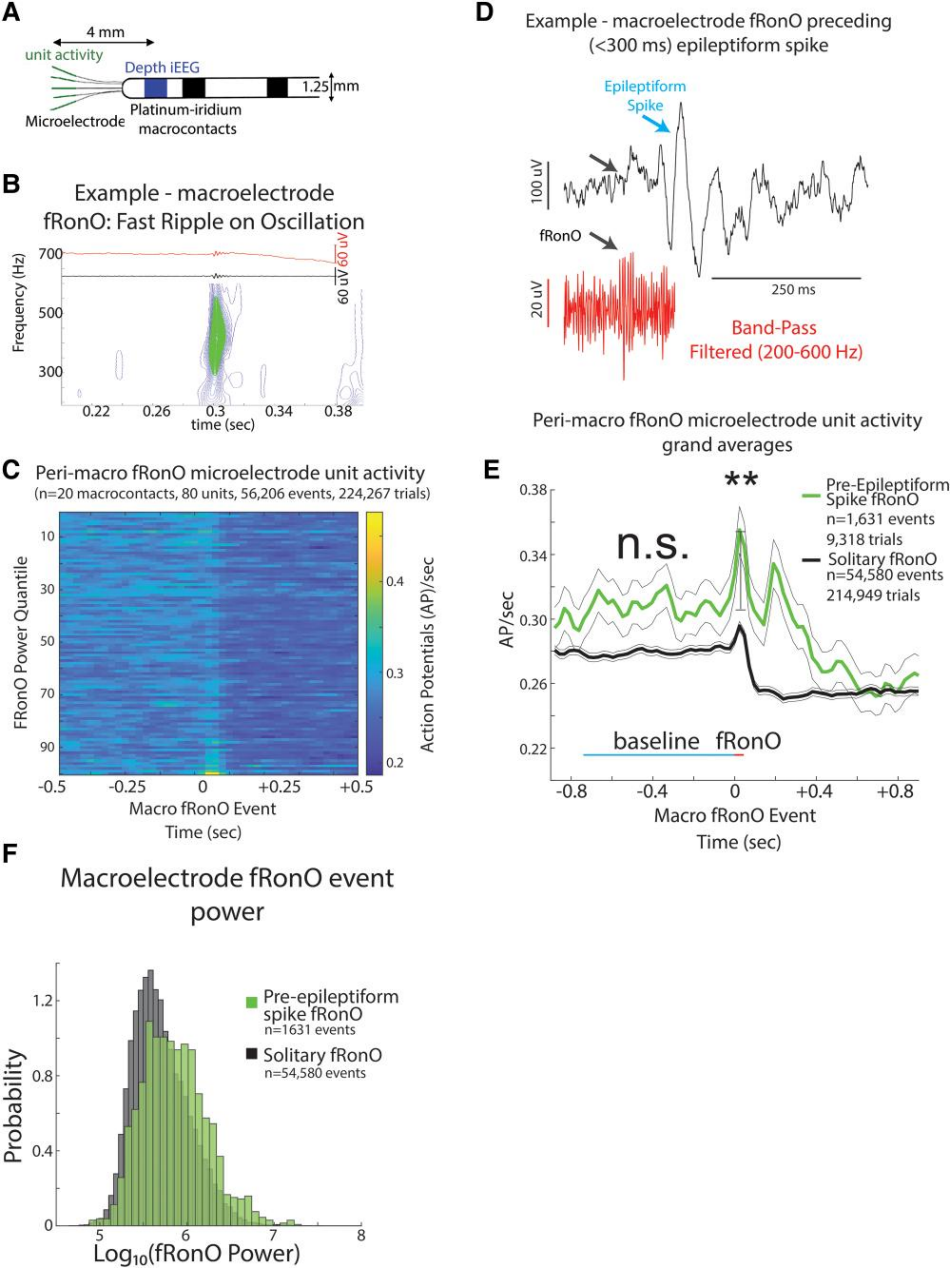
**iEEG fRonO preceding (<300 ms) an inter-ictal iEEG spike is associated with higher spectral power and higher unit firing rate.** (**A**) An illustration of a Behnke–Fried depth electrode. The HFO and spike events are detected in the iEEG recorded from the mesial most depth macroelectrode contact shown in blue, while the unit activity associated with these HFO and spike events is isolated from the LFPs recorded from the microelectrodes in green. Comparisons between the HFO and spike events and unit activity were made exclusively within a single Behnke–Fried depth electrode and not across different depth electrodes. (**B**) An example fRonO identified using the topographical analysis of the wavelet convolution that quantifies event power. (**C**) Peri-fRonO unit Gaussian smoothed AP firing rates from 80 units, which exhibited a significant fRonO triggered increase in firing (*P* < 0.001, FDR corrected), averaged by quantiles defined by the log_10_(power) of the fRonO events. Higher power fRonOs are associated with a greater increase in AP firing rate (GLMM, P≪1e−10, Supplementary Table 4). (**D**) Representative example of a fRonO event preceding a spontaneous epileptiform discharge with after-going slow wave in a macroelectrode recording. (**E**) Grand average of the Gaussian smoothed fRonO event-unit AP train trials for solitary peri-fRonO event trials not preceding an epileptiform discharge (black, 95% confidence interval), and peri-fRonO event trials that did precede (<300 ms) an epileptiform discharge (green). Differences in baseline (cyan line) firing rate were not significant (n.s. *P* > 0.05, GLMM, Supplementary Table 5). fRonO events that preceded an epileptiform discharge, relative to those that did not, had higher peak AP firing rates during the fRonO (red line) compared with baseline (cyan line) (**, GLMM, *P* < 1e−9, Supplementary Table 2), as well as an after-going secondary increase in firing rates. (**F**) Normalized histogram of fRonO event spectral power. fRonO events preceding spikes had a larger power (*t*-test, *P* < 1e−10, Cohen’s *d* = 0.475) than solitary fRonO, and the interaction of fRonO power with pre-spike status explained the increase in firing rates (GLMM) during fRonO preceding spikes (GLMM, *P* < 1e−5, Supplementary Table 4).

All the RonO, fRonO, RonS, or fRonS events detected in a single macroelectrode contact’s recording were individually compared with the corresponding APs from each individual unit isolated from the LFPs recorded from the bundle of microelectrodes distal to that macroelectrode (within <∼4 mm). Within each individual unit, for each macroelectrode HFO event, a trial was generated consisting of a 2 s raster centred at the onset of the HFO event with a resolution of 1 ms (Figs 1 and 2A). This raster was then convolved with a 100 ms Gaussian kernel, and the resulting AP train was down sampled to 40 Hz. We used a 100 ms kernel to better quantify HFO-related firing as opposed to examining fine temporal structure. For each HFO event-unit trial, the pre-event baseline unit firing rate was defined as the mean firing rate of the Gaussian smoothed AP train rate beginning 750 ms prior to HFO onset until the event (i.e. bl-fr). The peak HFO-unit firing rate was defined as the maximum of the Gaussian smoothed AP train rate during the duration of the HFO event (i.e. hfo-fr), and another value hfodiff-fr was defined as hfo-fr minus bl-fr.^17^ Since we wished to examine the factors that reliably modulate AP firing during HFO events, we excluded units from the model that did not significantly change in firing rates during the HFO events. Often this occurred because few HFO events were detected in the unit’s corresponding macroelectrode recording (Supplementary Fig. 1). Thus, a paired *t*-test was used to compare the bl-fr with the hfo-fr for each type of HFO event, across all trials, but within units. If the *P*-value exceeded 0.001 after controlling for the Holm–Bonferroni false discovery rate (FDR), the unit was excluded from the GLMMs analysis.

### Implementing GLMMs

The data for the GLMMs consisted of all the combined HFO event-unit AP train trials across all units, macroelectrodes and patients. For each category of HFO, the GLMMs fit the bl-fr or the hfodiff-fr using a Gaussian distribution, an identity link, a fixed intercept and the maximum quasi-likelihood method in Matlab using the fitglme.m function. Random effects included the unit identifier, macroelectrode contact identifier and patient identifier. All these identifiers were distinct (i.e. unit numbers did not repeat across different macro-microelectrode pairs). Fixed effect included the log_10_ (spectral power of the HFO event) recorded in the macroelectrode, and whether the RonO or fRonO preceded (<300 ms) an epileptiform spike or whether a RonS or fRonS followed (<300 ms) a RonO or fRonO in the macroelectrode recording, one GLMM used unit type (single unit or multi-unit) as a fixed effect. At most, each GLMM included only three fixed effects, including HFO power, with four interaction terms. The categorical neuroanatomical location of the unit was not used as a fixed effect because the unit identifier was included as a random effect.

### Identification of the SOZ and resected electrodes and related metrics

With respect to seizure outcome following surgery, Engel Class 1 refers to freedom from disabling seizures, Engel Class 2 to rare disabling seizures, Engel Class 3 to worthwhile improvement in seizures and Engel Class 4 to no worthwhile improvement. The epileptologist-defined SOZ contacts were aggregated across all seizures during the entire iEEG evaluation for each patient. The identification of the named electrode contacts in the resection cavity was performed manually in itk-SNAP (https://itk-snap.org) prior to performing calculations.^29^ The SOZ and resected ratio were calculated, within patients, as the number of HFO events detected from the SOZ or resected contacts, respectively, divided by the total number of detected HFO events. The FR rate-distance radius difference was calculated by first deriving the rate (events/min) of all fRonS events and the fRonO events with a peak spectral frequency >350 Hz per contact and then computing a rate-distance adjacency matrix as the average rate between contact pairs multiplied by the Euclidian distance (mm) between the contact pairs for all pair combinations >0. A second rate-distance adjacency matrix was computed this way but using only contacts in the resection margins. The charpath.m function (BCT, https://sites.google.com/site/bctnet/) was used to calculate the geometric radius of these scaled distance graphs represented by the rate-distance adjacency matrices, and the radius difference was defined as the √(radius of all electrodes − radius of the resected electrodes).^29^ A smaller radius difference corresponds to less residual FR activity. This measure was utilized because it accounts for spatial under sampling of FR generating tissue by the iEEG implant.

### Statistical methods

Paired and unpaired *t*-tests were performed in Matlab using ttest.m and ttest2.m; Cohen’s *d*ʹ for effect size was computed using computeCohen_d.m. Pertinent statistical methodological details are also included in the following sections: (i) ‘Preparing and implementing GLMMs’; and (ii) ‘Spike sorting and characterization of HFOs and epileptiform spikes’.

## Results

### Patient characteristics and isolation of electrophysiological events

To study the intricate relations between HFOs, inter-ictal spike,s and neuronal spiking activity, we combined microelectrode and macroelectrode data recorded during non-REM sleep, as well as post-surgical seizure outcome data in two separate patient cohorts. First, in six patients with medically refractory focal seizures who were implanted with depth electrodes, three were diagnosed with mesial-temporal lobe epilepsy (MTLE), one with MTLE and neocortical epilepsy, one with neocortical epilepsy and one with an unknown site of seizure onset. Each of the depth electrodes in these patients contained a bundle of eight microelectrodes extending beyond the distal tip that was positioned in mesial temporal lobe or medial neocortical structures (Fig. 2A).^27^ We isolated HFOs and sharp spikes in the iEEG and APs from 217 units in 175.95 iEEG contact-hours of synchronized macroelectrode and microelectrode recordings during N2/N3 sleep (Table 1). Second, to verify the findings in this small cohort of clinical significance, we isolated HFOs and sharp spikes in 1009.7 contact-hours of iEEG recordings during non-REM sleep in a separate cohort of 23 patients who underwent the same invasive EEG tests, but were not implanted with microelectrodes, and who subsequently had resections for their medically refractory focal seizures (Supplementary Table 1).^29^ Many of these patients had predictors for poor post-surgical seizure outcome, including six who had normal MRI, five had multilobar SOZs and six who were re-operated for a previous poor seizure outcome. Consequently, only 10 of these 23 patients were rendered seizure free (Supplementary Table 1).

**Table 1:** Clinical demographics and a full description of the brain structures and number of units identified in the recordings

Patient/age/gender	PET/MRI findings	Seizure onset zone (SOZ)	Resection/outcome	Macroelectrode/units
398/41/F	Metabolic and structural abnormalities left temporal. Pathology focal cortical dysplasia (FCD) IB	Left temporal spreading to right entorhinal cortex	Left temporal, significant improvement	L H: L A: L PHG: 6 units, L AC: 9 units, L TO: R H: 5 units, R A: R EC: 11 units, R PHG: 2 units, R AC: R TO:
406/33/F	Mild metabolic abnormality left temporal, structural abnormality right frontal lobe and left hippocampal T2 hyperintensity	Left medial temporal lobe	No surgery	L H: 6 units, L A: 8 units, L PHG: 13 units, L AC: 3 units, L SMA, R H: 8 units, R A: 8 units, R PH: 6 units, R AC: 4 units, R OF, R SMA
416/26/M	Mild metabolic abnormality right temporal, mild structural abnormality left temporal	Bilateral mesial temporal lobe and right inferior frontal gyrus	No surgery	L H: 3 units, L A: L EC: 1 unit, L AC: 3 units, L OF: 10 units, R H: 16 units, R A: 8 units, R EC: 7 units, R TG: 12 units, R AC: R OF: R IFG:
417/23/M	Metabolic abnormality right parietal lobe, normal structural. Pathology FCD IIA and IIB	Right parietal	Right parietal seizure free	L H: L A: L PHG: 4 units, L P: 8 units, R H: 9 units, R A:, R PH: 8 units, R P: 7 units
422/19/F	Volume loss due to prior left anterior mesial temoral lobectomy. Normal around margins of resection site and elsewhere	Not localized	No surgery	L PH: 8 units, L AC: 1 unit, L OF: 11 units, L SMA: 8 units, L PT: 2 units, R H: 5 units, R PH: 6 units, R OF: 7 units, R P: 10 units
423/52/F	Metabolic and structural abnormalities left temporal including amygdala T2 hyperintensity. Pathology gliosis	Left mesial temporal lobe	Left temporal seizure free	L H: 5 units, L A: L EC: 8 units, L OF: 7 units, R H: 1 units, R A: R EC: 11 units, R OF:

L, left hemisphere; R, right hemisphere; H, hippocampus; A, amygdala; EC, entorhinal cortex; PHG, parahippocampal gyrus; TG, temporal gyrus; AC, anterior cingulate; MC, middle cingulate; PC, posterior cingulate; OF, orbitofrontal and medial prefrontal cortex; SMA, supplementary motor area; *P*, parietal cortex; TO, temporo-occipital, PT, posterior temporal cortex; FG, fusiform gyrus; IFG, inferior frontal gyrus.

### fRonO and associated neuronal firing are increased when followed by inter-ictal spike

Based on results from our prior investigation of fRonO and epileptiform spikes, we defined fRonO and RonO preceding spikes as occurring within <300 ms.^26^ We defined iEEG spikes as either fRonS, RonS, or sharp spikes (no FR or ripple), which accounted for 19.8, 71.5 and 8.7% of all spikes, respectively. We compared peak firing during the fRonO and RonO events with a baseline epoch defined as beginning 750 ms before the events. We then asked if there was differential AP firing accompanying fRonO and RonO that preceded spikes versus solitary fRonO and RonO, i.e. without subsequent spike. Figure 1A provides a schematic illustration of the different categories used for this data analysis.

**Figure 3:**
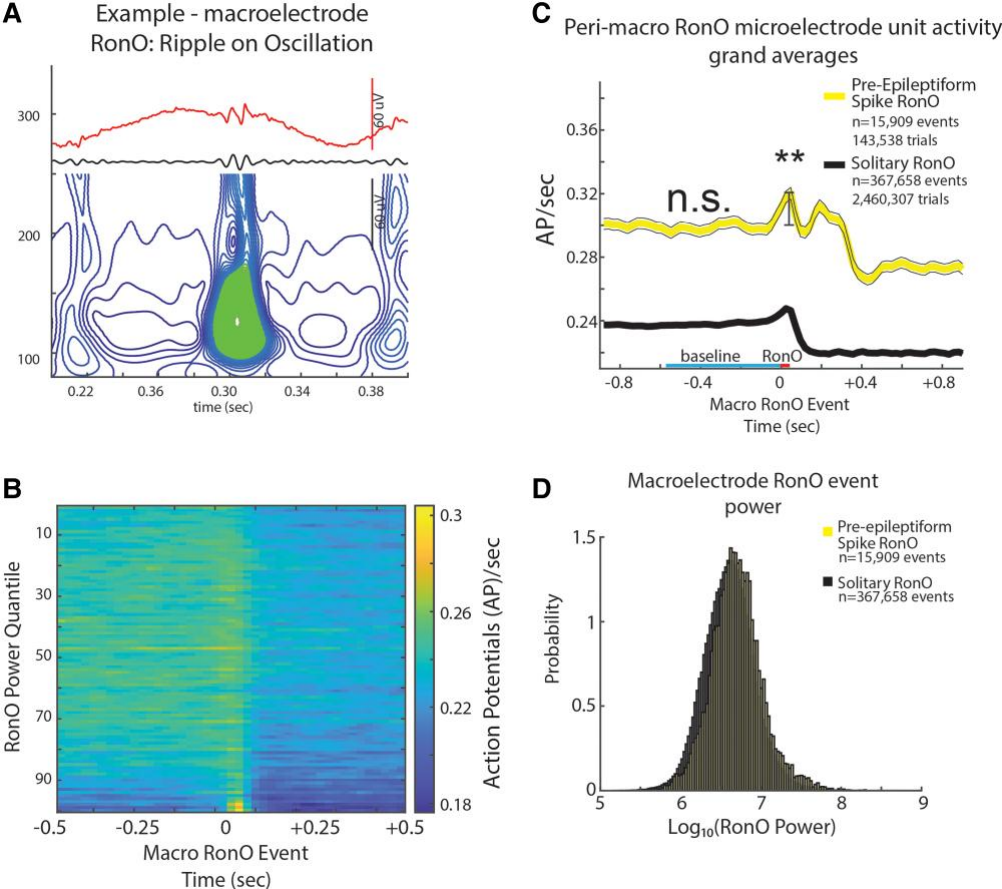
**iEEG RonOs associated increases in unit firing rate are larger if the RonO precedes (<300 ms) an epileptiform spike.** (**A**) An example RonO identified using the topographical analysis of the wavelet convolution. (**B**) Peri-RonO unit Gaussian smoothed AP firing rates from 174 units, which exhibited a significant RonO triggered increase in firing (*P* < 0.001, FDR corrected), averaged by quantiles defined by the log_10_(power) of the RonO events. Higher power RonOs are associated with a greater increase in AP firing rate (GLMM, P≪1e−10, Supplementary Table 7). (**C**) Grand average of the Gaussian smoothed HFO event-unit AP train trials for all peri-RonO event trials not preceding an epileptiform discharge (black, dotted lines = 95% confidence interval), and peri-RonO event trials that did precede (<300 ms) an epileptiform discharge (yellow). Although the baseline (cyan line) firing rate appears different between the two conditions, no increase was detected in the GLMM that accounted for RonO power as a fixed effect and the random effects of patient, macroelectrode and unit [not significant (n.s.), *P* > 0.05, Supplementary Table 9]. RonO events that preceded an epileptiform discharge, relative to those that did not, had an increased peak AP firing rate during the RonO event (red line) compared with baseline (cyan line) (**, GLMM, *P* < 1e−10, Supplementary Table 6). (**D**) Normalized histogram of fRonO event power in macroelectrode recordings. RonO events preceding spikes had a larger power (*t*-test, *P* < 1e−10, Cohen’s *d* = 0.3) than solitary RonO, and the interaction of RonO event power and pre-spike status significantly predicted increased firing rate (GLMM, *P* < 0.01, Supplementary Table 7).

Irrespective of coincidence with spikes, among the 217 units, 80 and 174 increased their firing rate during the fRonO and RonO (*t*-test, *P* < 1e−3, FDR corrected), respectively. None of the units decreased their firing rate during either fRonO or RonO. We found that peak unit firing rates, with respect to baseline (i.e. peak firing rate), were higher during pre-epileptiform spike fRonO (‘pre-Spike’, GLMM, *P* < 1e−9, Supplementary Table 2, Fig. 2B–E) and pre-Spike RonO (GLMM, *P* < 1e−10, Supplementary Table 6, Fig. 3A–C) when compared with solitary fRonO and RonO. Following solitary fRonO or RonO, peak firing rate decreased (Figs 2C, E and 3B, C). However, for pre-Spike fRonO or RonO, the Gaussian smoothed HFO event-unit AP train trials exhibited a second peak in firing rate following the HFO event (Figs 2E and 3C) corresponding to the after-going epileptiform spike. The baseline firing rate (−750 ms) prior to preSpike fRonO (Fig. 2E, Supplementary Table 5) was not significantly different from that of solitary fRonO (GLMM, *P* > 0.05). The baseline firing rate (−750 ms) prior to pre-Spike RonO (Fig. 3E, Supplementary Table 8) was significantly greater than solitary RonO (GLMM, *P* < 1e−10, Fig. 3C, Supplementary Table 8) but, after including RonO power as a fixed effect, pre-Spike RonO showed no difference in baseline firing rate (GLMM, n.s., Fig. 3C, Supplementary Table 9).

FRs are thought to be generated by AP population spikes,^38^ and during FRs, AP morphology may be altered.^39^ We compared spike trains of identified single units with identified multi-units during fRonO events. The multi-units were defined using more relaxed AP clustering that would be less susceptible to AP waveform morphology changes^27^ (see Supplementary Methods). We found that peak firing rates during a solitary fRonO event trended larger among the multi-units compared with single units (GLMM, *P* = 0.12, Supplementary Fig. 2, Supplementary Table 3). However, for the pre-Spike fRonO events, the peak firing was significantly larger among the multi-units when compared with the single units (GLMM, *P* < 1e−10, Supplementary Fig. 2, Supplementary Table 3). This indicates that single unit spike sorting underestimates the firing rate increase during fRonO and HFO spikes because of changes in AP morphology.

With respect to the power of the HFO events, fRonO preceding spikes had greater spectral power compared with solitary fRonO (*t*-test, *P* < 1e−10, Cohen’s *d* = 0.475, Fig. 2F). This was also the case for RonO (*t*-test, *P* < 1e−10, Cohen’s *d* = 0.3, Fig. 3D), but the effect size was smaller than fRonO. Using other GLMMs, that included both pre-Spike status and HFO power as fixed effects to fit the HFO associated peak firing rate, we found: (i) that fRonO power (Fig. 2C, GLMM, P≪1e−10, Supplementary Table 4) and RonO power (Fig. 3B, GLMM, P≪1e−10, Supplementary Table 7) both correlated with increased peak firing rate; (ii) the interaction between pre-Spike status and fRonO power (GLMM, *P* < 1e−5, Supplementary Table 4) or pre-Spike and RonO power (GLMM, *P* < 0.01, Supplementary Table 7) positively correlated and drove the correlation with peak firing rate; and (iii) the pre-Spike status alone exhibited a negative correlation with firing rate (fRonO, GLMM, *P* < 1e−4, Supplementary Table 4, RonO, GLMM, *P* < 0.01, Supplementary Table 7). Since fRonO power was increased for the pre-spike fRonO events, we analysed pre-spike fRonO power at latencies between 0 and 300 ms preceding the spike. We found fRonO of relatively greater power throughout this range of latencies but particularly for those fRonO that preceded spikes by <10 ms (Supplementary Fig. 3).

In summary, these results show that increased excitability is evident when a fRonO or a RonO precedes a spike and that this increased excitability is proportional to the power of the fRonO and RonO event.

### Inter-ictal fRonS and RonS associated neuronal firing are decreased when preceded by a fRonO

**Figure 4:**
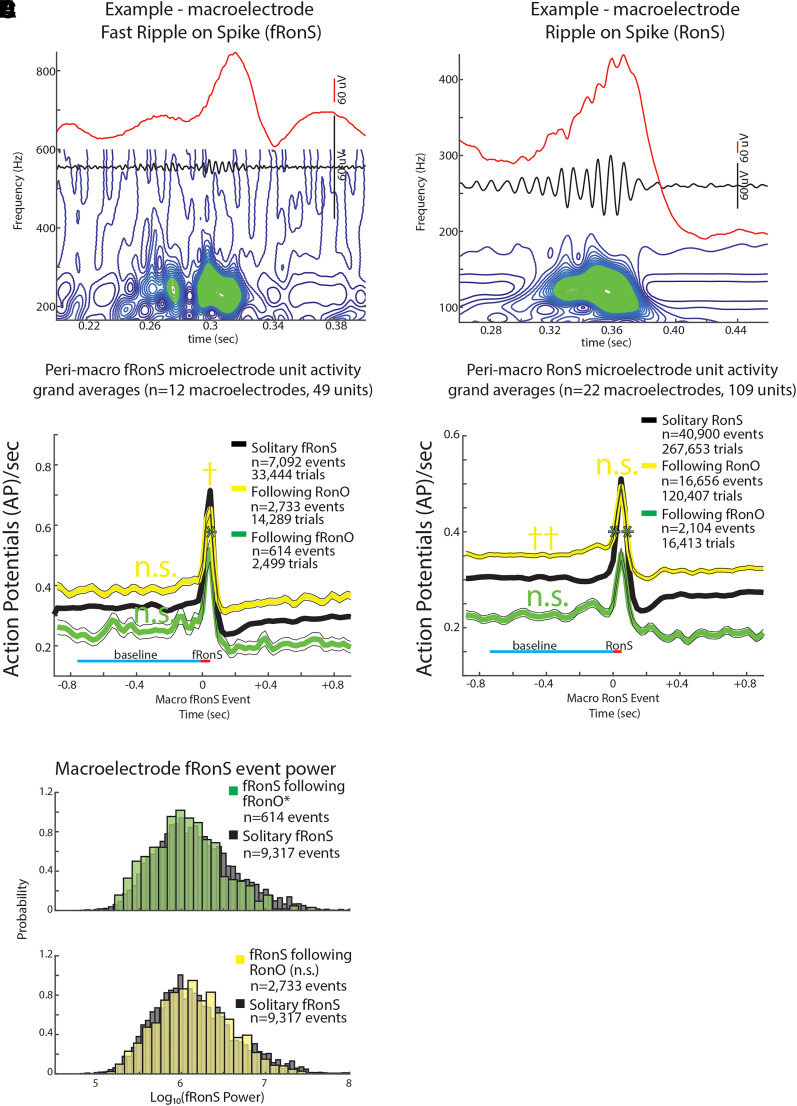
**Unit firing rates prior to and during fRonS and RonS are decreased if preceded (<300 ms) by a fRonO.** An example fRonS (**A**) identified using the topographical analysis of the wavelet convolution. (**B**) Grand average of the Gaussian smoothed HFO event-unit AP train trials peri-fRonS from 49 units, which showed statistically significant increased fRonS triggered firing (*P* < 0.001, FDR corrected). Shown are trial averages with no preceding event (black solitary, neither RonO nor fRonO), when a fRonS followed a RonO (yellow), or when a fRonS followed a fRonO (green). AP firing rate during the fRonS was proportional to fRonS log_10_(power) (GLMM, P≪1e−10, not shown, Supplementary Table 11). The baseline AP firing rate (cyan line) for fRonS that followed a fRonO or a RonO was not significantly different (n.s.) from solitary fRonS (n.s., GLMM, *P* > 0.05, Supplementary Tables 12 and 13). During the fRonS event (red line), the peak AP firing rates relative to baseline (cyan line) was decreased when fRonS event followed a fRonO (*, GLMM, *P* < 1e−4, Supplementary Tables 10 and 11) but increased if the fRonS event followed a RonO (†, GLMM, *P* < 1e−5, Supplementary Tables 10 and 11). The latter effect derived in the GLMM is not appreciated in the grand average shown in (**B**). (**C**) Normalized histogram of fRonS event power in macroelectrode recordings. fRonS power was reduced when it followed a fRonO (*t*-test, *P* < 1e−5, Cohen’s *d* = 0.21) but unchanged if the fRonS followed a RonO (*P* > 0.05). (**D**) An example RonS identified using the topographical analysis of the wavelet convolution. (**E**) Grand average of the Gaussian smoothed HFO event-unit AP train trials rates peri-RonS from 109 units, which showed statistically significant increased RonS triggered firing (*P* < 0.001, FDR corrected) as in (**B**). The baseline AP firing rate (cyan line) for RonS that followed a fRonO or RonO was not significantly different (n.s., GLMM, *P* > 0.05, Supplementary Table 16) or greater (††, GLMM, P≪1e−10, Supplementary Tables 16 and 17) than solitary RonS, respectively. During the RonS event (red line), the peak AP firing rates, relative to baseline (cyan line), was proportional to RonS log_10_(power) (GLMM, P≪1e−10, not shown, Supplementary Table 15); RonS events that followed fRonO, but not RonO (n.s.), exhibited decreased peak AP firing rates, relative to baseline, compared with solitary RonS (**, GLMM, *P* < 0.05, Supplementary Tables 14 and 15).

In this analysis, we shifted from fRonO/RonO aligned trials of AP trains to HFO spike (i.e. fRonS/RonS) aligned trials (Fig. 1B). We analysed peak AP firing during fRonS (Fig. 4A) or RonS (Fig. 4D), when compared with the 750 ms pre-event baseline epoch (i.e. peak firing rate), and asked whether the extent of AP firing was different when fRonS/RonS was preceded (<300 ms) by fRonO or RonO than when fRonS/RonS occurred alone (i.e. solitary, Fig. 1B). Of the 217 units, irrespective of coincidence with fRonO or RonO, 49 and 109 increased their firing rate during a fRonS and RonS, respectively (*t*-test, *P* < 0.001, FDR corrected). In contrast to a prior study of HFO spikes,^17^ no units decreased their firing rate.

In this analysis, we examined firing rate changes relative to the onset of the HFO superimposed on the spike and not the spike onset. Since the firing rate during an HFO spike may also be modulated by the epileptiform spike itself, or other drivers, we first generated trial-wise histograms of the time of peak firing of all the HFO spike event-unit trials relative to spike-HFO onset (Supplementary Fig. 4). We found that, among the HFO-unit Gaussian smoothed firing rate trials, maximal AP firing typically occurred at 0–20 ms after HFO spike onset. However, maximal AP firing also occurred at a frequency greater than baseline up to 60 ms before the HFO spike onset.^17^ The error in our calculations attributable to these trials was minimized by the use of Gaussian kernel smoothing.

With respect to identifying the influence of HFOs preceding HFO spikes, we found that during fRonS following fRonO (GLMM, *P* < 1e−4, Fig. 4A and B, Supplementary Table 10) and during RonS following fRonO (GLMM, *P* < 0.05, Fig. 4D and E, Supplementary Table 14), the peak firing rates were reduced in comparison with solitary fRonS and RonS. The baseline firing rates prior to fRonS following fRonO (GLMM, *P* > 0.05, Fig. 4B, Supplementary Table 12) and RonS following fRonO (GLMM, *P* > 0.05 Fig. 4E, Supplementary Table 16) were not significantly different from solitary fRonS and RonS.

Spectral power was lower for fRonS following fRonO (*t*-test, *P* < 1e−5, Cohen’s *d* = 0.21, Fig. 4C). To better understand this effect, we examined fRonS that followed fRonO power between 0 and 300 ms and found that fRonS power was only decreased for events that occurred at latencies >10 ms (Supplementary Fig. 5). When fRonS and RonS power were included as an additional fixed effect in GLMMs to fit peak firing rate during the events, the GLMM showed that: (i) among all fRonS and RonS, irrespective of preceding fRonO/RonO, higher peak firing rates correlated with higher fRonS power (GLMM, P≪1e−10, Supplementary Table 11) and RonS power (GLMM, P≪1e−10, Supplementary Table 15); (ii) the decrease in peak firing rate in fRonS following fRonO, relative to solitary fRonS, was due to the following fRonO status alone (GLMM, *P* < 0.02, Supplementary Table 11) and not the fRonS power interaction (GLMM, *P* = 0.05, Supplementary Table 11); and (iii) the decrease in peak firing rate for RonS following fRonO, relative to solitary RonS, was due to the interaction of following fRonO status with RonS power (GLMM, *P* < 0.01, Supplementary Table 15), whereas following fRonO status alone was positively correlated with increased peak firing rate during the RonS (GLMM, *P* < 0.01, Supplementary Table 15). However, RonS spectral power was unchanged for RonS following fRonO compared with solitary RonS (*t*-test, *P* < 0.05).

In summary, results show that fRonS and RonS that followed fRonO show a decreased peak firing rate when compared with solitary fRonS and RonS. However, when we examined fRonS that followed RonO, we found that they exhibited an increased peak HFO firing rate (GLMM, *P* < 1e−5, Fig. 4A and B, Supplementary Tables 10 and 11) when compared with solitary fRonS. Additionally, while fRonS following fRonO exhibited decreased power, fRonS following RonO exhibited no change in power (*t*-test, *P* > 0.05, Fig. 4C). RonS that followed RonO exhibited no significant difference in peak firing rate when compared with solitary RonS (GLMM, *P* > 0.05, Fig. 4D and E, Supplementary Table 14). With regards to baseline firing rate, we found no significant differences between fRonS following RonO and solitary fRonS (GLMM, *P* > 0.05, Fig. 4A and B, Supplementary Tables 12 and 13). However, RonS that followed RonO had a significantly higher baseline firing rate (GLMM, *P* > 0.05, Fig. 4D and E, Supplementary Tables 16 and 17) than solitary RonS.

### fRonO preceding inter-ictal spikes demarcate a SIZ in epileptogenic tissue

**Figure 5:**
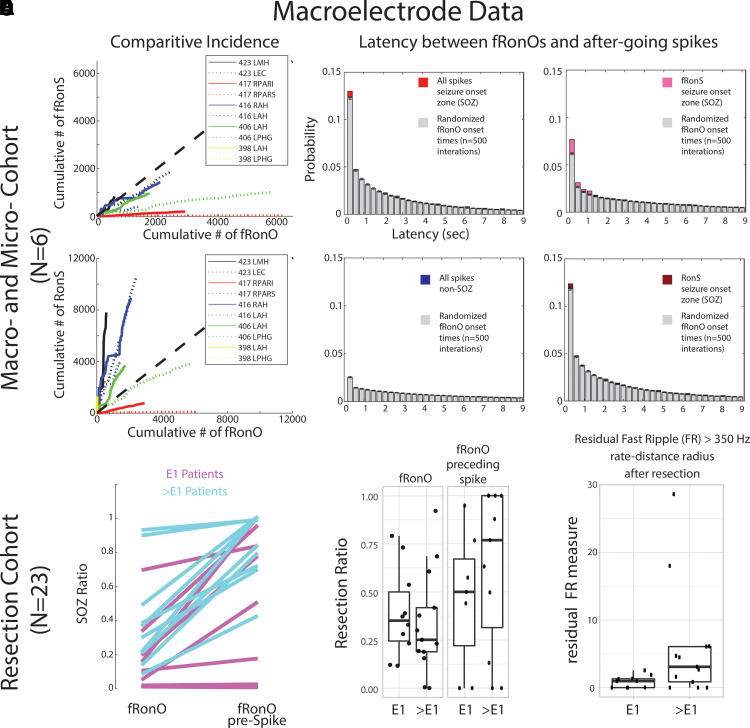
**fRonO preceding (<300 ms) epileptiform spikes occur mostly in the SOZ; however, resection of cortical territory generating fRonO with after-going spikes does not correlate with seizure freedom.** In the paired macroelectrode and microelectrode cohorts, (**A**) comparative incidence of fRonO with (fRonS, top), as well as fRonO with (RonS, bottom) in 10 macroelectrode channels in the SOZ calculated in bins of 60 s over the recording duration. Note that for most channels, multiple fRonOs occur before a fRonS is detected. However, RonS are generated in greater number than fRonO. (**B**) Normalized histogram of the latency between spike onset and fRonO in individual iEEG channels when computed using all spikes in the SOZ (red bars, *n* = 41 861 fRonO), all spikes in the non-SOZ (blue bars, *n* = 52 136 fRonO), only fRonS in the SOZ (pink bars) and only RonS in the SOZ (dark red bars). Latencies computed with boot-strapping statistics (see ‘Materials and methods’ section, *n* = 500 surrogates, grey bars) indicated that fRonO in the <300 ms bin exceeded chance (*z*-score = 4.61, *P* < 1e−5) but were less than chance in the non-SOZ (*z* = −7.72, *P* > 0.05, blue). The most significant effect was seen for fRonO with after going fRonS (*z* = 11.61, P≪1e−10). (**C–E**) Data taken from a cohort of 23 patients with stereo-EEG recordings, but no microelectrodes placed, who underwent resections. (**C**) The within-patient differences in the proportion of events in the SOZ for fRonO events compared with fRonO events preceding (<300 ms) spikes for patients with a seizure-free Engel 1 (E1) outcome (magenta) and non-seizure-free patients (cyan, >E1, Engel 2–4). The proportion of fRonO events preceding spikes in the SOZ was significantly higher (rank sum, *P* < 1e−5). (**D**) Box plots of the ratio of fRonO (left) and fRonO preceding spike (right) events within the resection margins stratified by post-operative seizure outcome. (**E**) A spatial graph theoretical measure, the FR rate-distance radius resection difference, that quantifies both the volume and activity of the residual FR generating tissue remaining after resection that was significantly decreased in seizure-free patients (rank sum, *P* < 0.05). L, left hemisphere; R, right hemisphere; AH/MH, anterior, middle hippocampus; EC, entorhinal cortex; PAR, parietal (sup./inf.); PHG, parahippocampal gyrus.

**Figure 6:**
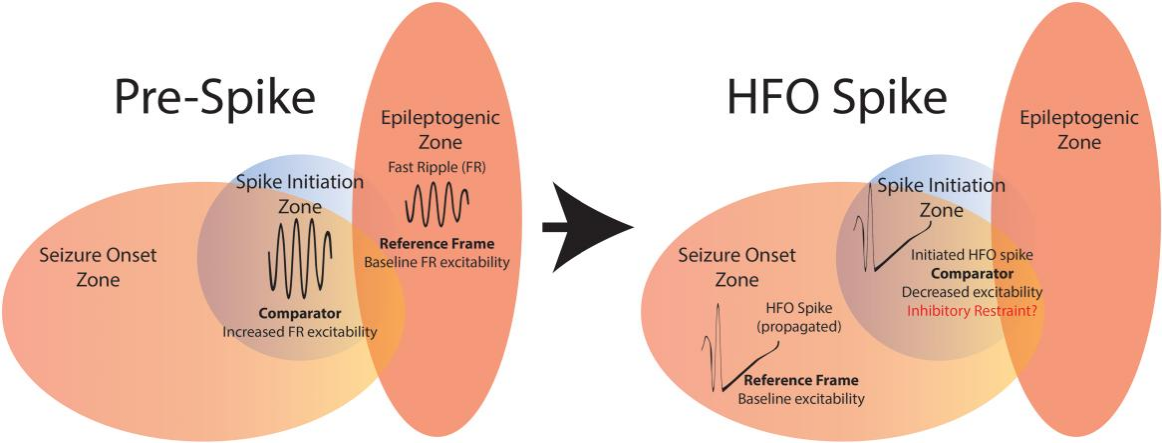
**Schematic of the summary of findings.** Resecting fRonO preceding spikes did not result in seizure freedom; thus the SIZ (blue) does not always strongly overlap with the EZ (red). However, fRonO preceding spikes were found in greater proportions in the SOZ, so the SIZ does strongly overlap with the SOZ. Prior to spike initiation (right), a fRonO in the SIZ is generated with increased single unit firing rate relative to a smaller power fRonO in the EZ. Within 300 ms of the fRonO occurring in the SIZ, a fRonS or RonS is generated in the SIZ. This suggests the fRonO may prime the fRonS or RonS (HFO spike, right). Unit firing rate during a fRonS or RonS in the SOZ, outside the SIZ, is relatively more robust compared with the unit firing rate during the fRonS or RonS in the SIZ. The decreased excitability in the SIZ may be due to an inhibitory restraint mechanism that ordinarily prevents fRonS or RonS initiation.

Thus far, we found increased excitability during fRonO and decreased excitability during the ensuing fRonS and RonS. Next, we quantified where fRonO and fRonS/RonS occur and whether the location might correspond with a SIZ. We assumed that if fRonO primes fRonS or RonS, then the rate of fRonO should exceed the rate of fRonS or RonS in the SIZ. To test this, we calculated the ratio of fRonS to fRonO and RonS to fRonO occurrences in 1-min bins in the SOZ. We found the ratio of fRonS to fRonO was less than 1 but RonS to fRonO was greater than 1 (Fig. 5A). We next examined whether fRonO preceding fRonS or RonS occurred more often than chance using boot strapping (see ‘Materials and methods’ section). In the SOZ, the probability of a fRonO preceding fRonS or RonS (<300 ms) was greater than chance (*n* = 500 surrogates, *z*-score = 4.61, *P* < 1e−5, Fig. 5B), but in the non-SOZ, fRonO preceding fRonS or RonS was less than chance (*z* = −7.72, *P* < 1e−5, Fig. 5B). Specifically, in the SOZ, greater differences between the observed values and boot strapped-surrogates were found with fRonO preceding fRonS (*z* = 11.61, P≪1e−10, Fig. 5B) than the differences for fRonO preceding RonS (*z* = 3.63, *P* < 1e−4, Fig. 6B). In contrast to fRonO, in the SOZ and non-SOZ, RonO preceding fRonS or RonS occurred less than chance (*z* = −15.44, *P* < 1e−10 and *z* = −7.75, *P* > 0.05, respectively). Collectively, these results indicate fRonO and not RonO likely prime spikes, especially fRonS in a SIZ.

Extending the results from the preceding paragraph, we assessed fRonO preceding all spikes (fRonS, RonS, sharp spikes) in relation to the clinical SOZ and seizure outcome in a separate cohort of 23 patients who underwent resections. For each patient, we computed the SOZ ratio defined as the proportion of fRonO preceding spikes on macroelectrode contacts in the SOZ contacts to the total macroelectrode contacts in SOZ and non-SOZ. The SOZ ratio of fRonO preceding spikes was greater than the SOZ ratio of solitary fRonO (rank sum, *P* < 1e−5, Fig. 5C), but fRonO preceding spikes were not identified in five patients. In addition, across all patients, the proportion of fRonO preceding spikes in the SOZ was 66.5% and only slightly greater than solitary spikes at 64.5%. These results suggest that fRonO preceding spikes and solitary HFO spikes and sharp spikes, but not solitary fRonO, occur mostly in the SOZ.^40^

Theoretically, resection of fRonO preceding spikes should correlate with good seizure outcome because it is found in high proportions in the SOZ. We found that the mean proportion of fRonO events recorded from contacts within the resection margins relative to the total number of fRonO events recorded from all the contacts (resection ratio) was higher across seizure-free patients compared with those who were not seizure free. However, when we calculated these same resection ratios using fRonO preceding spikes, the mean resection ratios were paradoxically lower in seizure-free patients than those who were not seizure free, though differences were not statistically significant (rank sum, *P* > 0.05, Fig. 5D). Also, in all patients, we found a higher resection ratio of fRonO preceding spikes than solitary fRonO (sign rank, *P* < 0.05, Fig. 5D), indicating that fRonO preceding spikes to a greater extent than solitary fRonO demarcate the clinical SOZ that was targeted for resection.

Since neither of the two fRonO resection ratios was significantly greater in the seizure-free patients, it raises the issue of whether FRs are satisfactory biomarkers of epileptogenic tissue. To examine this further, we used a spatial graph theoretical measure of the residual FR rate-distance radius following a resection. This measure uses a combination of fRonS and fRonO >350 Hz rates (events/min) and weighs those by the spatial distance between the generator sites to compensate for spatial under-sampling by the SEEG implant. Using this measure, we found significantly lower residual FR rate-distance in the 10 seizure-free patients (rank sum, *P* < 0.05, Fig. 5E). Because our results show that the SIZ and SOZ^29^ had been resected in most of the subjects overall, but low levels of FR-generating tissue were found primarily in the seizure-free subjects; it can be concluded that while the SIZ is epileptogenic, epileptogenic regions can be found outside the SIZ and SOZ and generate solitary fRonO.

## Discussion

Bridging prior work demonstrating that HFOs preceding spikes have increased neuronal firing rates^41^ and propagating fRonO increases the probability of an after-going spike,^26^ this study shows that the coincidence of fRonO preceding (<300 ms) spikes is statistically more common than chance and that fRonO preceding spikes correspond with increase neuronal excitability, which could help generate an ensuing spike. We also found during the spike, neuronal firing was reduced, which could associate with an inhibitory restraint that has been demonstrated in preventing inter-ictal spike propagation.^42–45^ We extrapolated on these mechanistic findings in a larger cohort of patients who underwent resections and found that fRonO preceding spikes were generated in regions defined as the epileptogenic SOZ by the clinician that were subsequently resected, which supports fRonO preceding spikes demarcating a SIZ.^9,40^ However, resecting the SIZ did not correlate with seizure-free post-operative seizure outcome, which suggests that epileptogenic regions are necessary but not sufficient for spike generation.

### Unit firing rate properties that underlie increased excitability changes during spike priming

A novel finding of our study was the strong correlation between increased spectral power of HFO and corresponding peak firing rates during the HFO. The HFO spectral power can reflect the recruitment of a larger pool of neurons for HFO generation in pathological regions,^46^ although measurements of spectral power are confounded by differences in the distance from the generator to the electrode contact.^47^ The pre-Spike fRonO and RonO exhibited higher associated firing rates than solitary fRonO and RonO, while the corresponding spectral power of these pre-Spike fRonO and RonO events was also much greater than the solitary fRonO and RonO events. This suggests that macroscale recruitment of pathological neural circuits is required to generate pre-Spike fRonO and RonO. Distinguishing putative excitatory from inhibitory single units may be helpful to investigate pre-Spike fRonO and RonO, but a prior investigation of the single unit correlates of epileptiform discharges found no difference in the firing properties of excitatory and inhibitory single units.^7^ Future experiments utilizing experimental epilepsy models with calcium or voltage imaging paired with neurophysiological recordings would be helpful to investigate pre-Spike fRonO or RonO mechanisms. Additionally, a larger study is required to investigate whether, in the SOZ/EZ, unit firing rates are more robust during solitary RonO and fRonO; however, it is already established that in the SOZ/EZ, HFO power is larger.^46,48^

### Unit firing rate properties that may underlie inhibitory restraint after spike priming

We observed that the mean firing rate of the grand average of all fRonS and RonS event-unit AP trains more than doubled from baseline during the fRonS/RonS. However, the overall baseline firing rates were lower than that reported in similar investigations.^7,17^ The most likely explanation is our inclusion of single units and use of a Gaussian kernel and a peak firing measure to estimate AP trains, which may also explain why we did not identify units that decreased in firing during HFO spike.^7,17^ In contrast, Guth *et al*.,^17^ calculated each unit’s firing rate in 20 ms bins and then used a cluster analysis with boot strapping to examine HFO spike-related increases and decreases. This method is more accurate than the Gaussian kernel methodology. However, it is not well suited to measure complex spike train time courses in response to paired events, at variable latencies. Importantly, all the studies of the firing rate during inter-ictal discharges measured from *in vivo* recordings in patients show that they are substantially less than that reported in human cortical slices^49^ and much less than in chemoconvulsant-treated animals.^50,51^

We found when a fRonS/RonS followed a fRonO AP peak firing was decreased at the time of the HFO spike. We also observed, but did not quantify, that following the generation of all the HFO types, unit firing rate decreased for at least 500 ms. A recent study found that following fRonS generation, a decrease in firing rates is seen even spatially far from the generator.^52^ Thus, it is possible that decreased excitability during a fRonS/RonS is a result of the preceding fRonO alone. This is unlikely however because when a fRonS or RonS followed a fRonO, there was no change in the baseline firing rate. One interpretation is that decreased excitability during HFO spikes that follow fRonO results from an inhibitory restraint mechanism initiated during or just after priming. Inhibitory restraint of spike propagation has already been well established in animal models,^42–45^ but its importance in spike initiation is less clear.

In contrast to the spikes following fRonO with relatively decreased excitability, the spikes following RonO exhibited either increased or no change in excitability. Furthermore, fRonO preceded HFO spikes more often than chance, whereas RonO preceded HFO spikes less than chance. Thus, fRonO events are a better candidate for promoting HFO spikes than RonO.

### The role of fRonO propagation in spike priming

We previously found that fRonO that propagates in the SOZ is strongly coupled to the DOWN state and is more often followed by epileptiform discharges than non-propagating fRonO. In this case, the fRonO precedes the epileptiform spikes by <10 ms.^26^ Here, we could not relate fRonO propagation to unit firing activity because iEEG recordings were only available from the most mesial electrodes. However, we found that fRonO that precedes epileptiform spikes by <10 ms may be distinct from fRonO that precedes spikes by 10–300 ms (Supplementary Figs S3 and S5). Thus, based on the current results, we can elaborate on our past results and propose a fRonO-spike complex, in which the fRonO precedes the spike by <10 ms, and can propagate together, but an earlier event >10 ms may be required to prime this complex to occur first. Future efforts will also examine slow wave modulation of excitability during RonO/fRonO and how this modulation influences after-going spikes. In contrast to our past work,^37^ here we did not distinguish HFOs on oscillations by oscillation sub-type (slow, delta, theta, spindle) because we focused on the coincidence of HFO events.

### Epileptogenic regions are necessary but not sufficient for spike priming

The clinical gold standard for planning resective epilepsy surgery is electroclinical correlation and removal of the SOZ.^53^ In our clinical cohort, we found that fRonO preceding spikes were almost always generated in the SOZ, which, according to our analysis of the unit activity corresponding to the paired events, indicates that the SIZ strongly overlapped with the SOZ (Fig. 6). Moreover, only in the SOZ, fRonO precedes spikes at rates greater than chance. Past work has shown that spikes following gamma oscillation (30–100 Hz) bursts are more exclusively generated in either the SOZ or in resected regions in seizure-free patients than spikes alone.^54,55^ It is likely that fRonO priming is not the sole mechanism for spike initiation, and gamma oscillations, that occur very frequently, also play a role and may generate different types of spikes.

In addition to fRonO preceding spikes occurring primarily within the SOZ, fRonO preceding spikes were also mainly generated at sites within the resection margins, in both the patients who were rendered seizure free by surgery and the patients with failed surgery. This implies that for the patients with failed surgeries, other epileptogenic regions outside of the SOZ and SIZ could promote seizures after the surgery. Our data show that this other region, the residual EZ,^13,53,56^ maybe identified using spatial estimates of residual FR generation (Fig. 6),^29^ and such a region may generate solitary fRonO and seizures beyond its boundaries. Our results do not imply that the SIZ can be left intact and a seizure-free outcome still achieved. To the contrary, our past investigation of intra-operative iEEG recorded from subdural electrodes in temporal lobe epilepsy patients found that unresected fRonS,^22^ a strong biomarker of the SIZ, correlates with failed surgery. In addition, earlier studies had found that patients with well-localized spikes^57,58^ and spikes that post-operatively strongly decreased in occurrence, are more likely to be seizure free.^57–59^ However, intra-operative electrocorticography, which involves resecting regions using spike rates measured in subdural electrodes alone, does not always result in seizure freedom, even when a combination of ripple and FR is used instead.^60,61^

A limitation of our study is that comparing the unit activity in the microelectrodes with the HFOs and spikes in the iEEG recorded by the most proximal macroelectrode may be inaccurate, because the macroelectrode may be positioned outside the neuroanatomic structure, or even lesion, the microwires are recording from. Additionally, the epileptic network that initiates seizures (i.e. SOZ) may be proximal to the macroelectrode but distal to the microelectrode. A confound of our study is that the features that distinguish physiological RonO and pathological RonO are not well established.^62^ Potentially, a subpopulation of the RonO events recorded in the iEEG of the hippocampus could be physiological sharp wave ripple events. Yet, in contrast to RonO, the iEEG fRonO events are unlikely to be physiological in origin.^63^ A further limitation of our study is that we did not use the temporal ordering of epileptiform spikes to identify the SIZ because it required distinct methods for isolating bluntly contoured discharges. Two studies implementing this method similarly localized the SIZ to epileptogenic regions,^9,40^ although this was not the case in another study utilizing microelectrode arrays instead of macroelectrodes.^8^ It will be necessary in the future to combine our study’s fRonO and spike coincidence analysis with an iEEG spike source localization analysis to determine if the location of the SIZ is agreed upon.

## Conclusion

We show that the generation of a fRonO, reflecting increased neuronal excitability, primes the generation of an after-going spike within an epileptogenic region. Additionally, during the initiation of this subsequent spike, excitability is reduced relative to solitary spikes. This likely reflects an inhibitory restraint that is well established for preventing the spatial propagation of spikes. In a clinical context, our results show that epileptogenic regions are necessary but not sufficient for spike generation. This is important in current practice because strategies to localize and resect just the SIZ or SOZ alone may not always succeed because of the possibility of a residual EZ. In future practice, it may be possible to prevent both spikes and seizures by pharmacologically or electrically inhibiting the occurrence of fRonO.

## Supplementary Material

fcad242_Supplementary_Data

## Data Availability

The data and code to reproduce the results and figures are available at https://zenodo.org/record/8125756 and https://github.com/shenweiss/FRexcitabilityprespike. The raw data are available upon reasonable request.
