# Supplementary material for: Fast ripples reflect increased excitability that primes epileptiform spikes
Source: Brain Commun. 2023 Sep 8;5(5):fcad242. doi: 10.1093/braincomms/fcad242 (PMC10587774; doi:10.1093/braincomms/fcad242)
Supplement: fcad242_Supplementary_Data [file fcad242_Supplementary_Data.pdf]

Supplementary Materials

Fast ripples reflect increased excitability that primes epileptiform spikes.

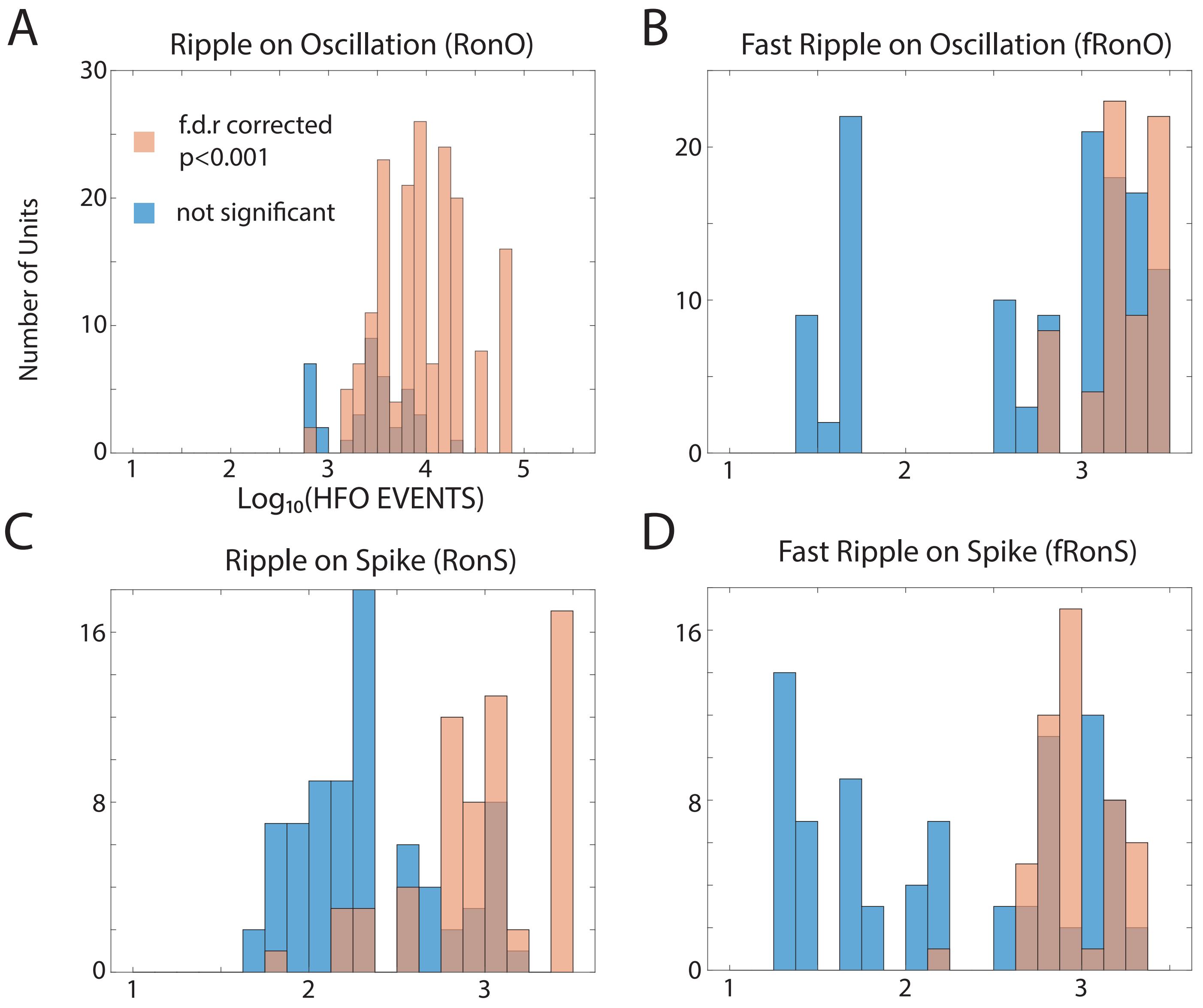

Supplementary Figure 1: Units with significant increases in peak gaussian smoothed firing rate during high-frequency oscillation (HFO) events exhibit higher HFO event rates. Histograms of the number of units showing significant (f.d.r. corrected $p<0.001$) increases in peak firing rate in red and number of units with insignificant increases in peak firing rate (blue) as a function of the log10 transformed number of HFO events recorded by the proximal macroelectrode contact for ripples on oscillations (A, RonO), fast ripples on oscillations (B, fRonO), ripples on spikes (C, RonS), fast ripples on spikes (D, fRonS). Note that some units showed insignificant increases in HFO related firing (insignificant) despite relatively high HFO counts. For fRonO 57% of the multi-units were insignifact, whereas 68% of the single units were insignificant. Abbreviations: RonO=ripple on oscillation; fRonO=fast ripple on oscillation; RonS = ripple on spike; fRonS = fast ripple on spike.

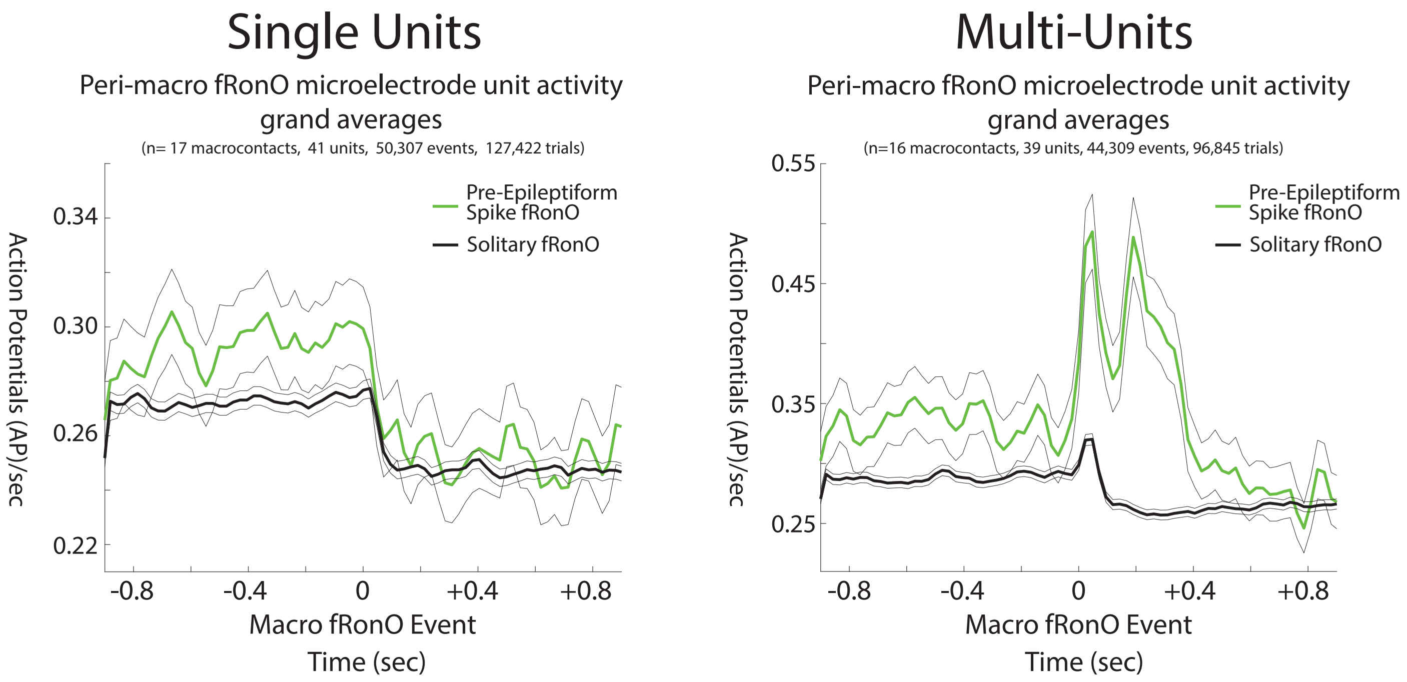

Supplementary Figure 2: Solitary and pre-epileptiform spike fast ripple on oscillation (fRonO) related increases in peak firing above baseline are greater in microelectrode recordings sorted as multi-unit activity (right) as oppossed to those sorted as single-unit activity (left). In addition the increase in firing rate corresponding to the after-going epileptiform spike are present in the multi-unit activity (right) but absent in the single-unit activity (left). Abbreviations: sec= second; macro= macroelectrode; AP= action potential; fRonO= fast ripple on oscillation

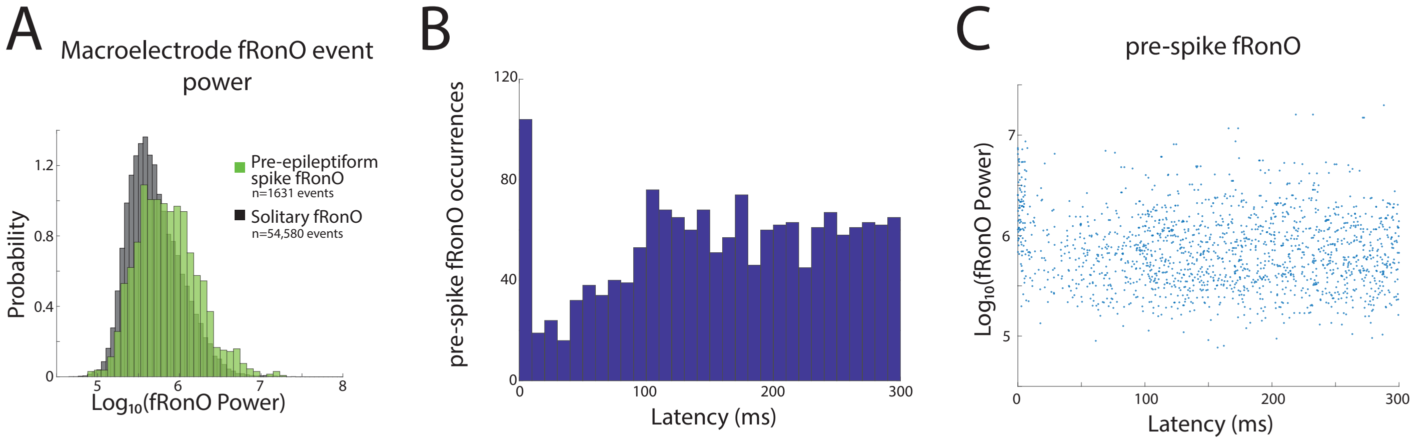

Supplementary Figure 3: An examination of pre-epileptiform spike fast ripple on oscillation (fRonO) power with increased temporal resolution. (A) Normalized histogram of fRonO event spectral power. fRonO events preceding spikes had a larger power (t-test, p<1e-10, Cohen's d=.475) than solitary fRonO. (B) Histogram of the latency in milliseconds (ms) between pre-spike fRonO and the after-going spike. Note that a relatively small, but distinct, subpopulation of events occurred within <10 msec of the spike. (C) Plot of the pre-spike fRonO power as a function of latency before the spike. Note that relatively larger powers (see A) were seen across a broad range of latencies. However, the power distribution for fRonO events occurring <10 ms before spikes exhibits relatively higher powers than the distribution of fRonO events with a latency >10 ms. Abbreviations: fRonO=fast ripple on oscillation;
ms=millisecond.

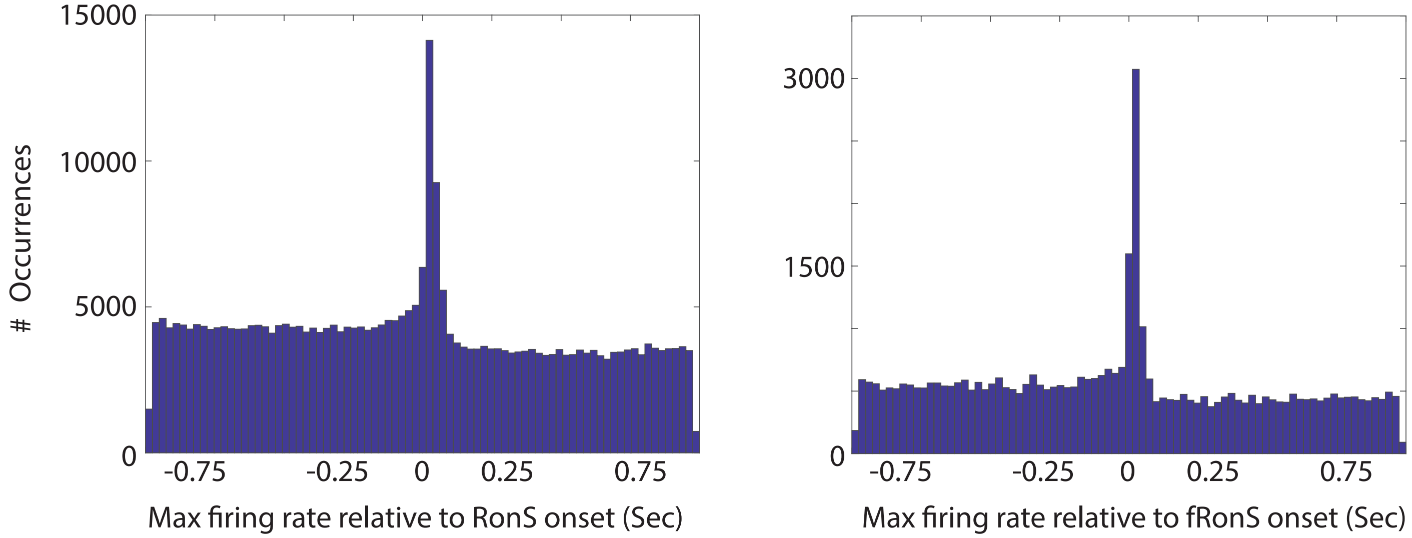

Supplementary Figure 4: A trialwise comparison of the latency of the maximum Gaussian smoothed unit firing rate relative to ripple (RonS) or fast ripple on spike (fRonS) onset. Shown is a histogram of the number of RonS (left) and fRonS (right) event-unit trials at each measured peri-event latency. Note that most frequently RonS/fRonS event-unit trials fire maximally at the time of RonS/fRonS onset or within 20 msec after the onset. However, probability of maximal firing is also higher than baseline at 10 msec before RonS/fRonS onset, and marginally higher up to 60 msec before RonS/fRonS onset. Abbreviations: Sec=second; RonS: ripple on spike; fRonS: fast ripple on spike.

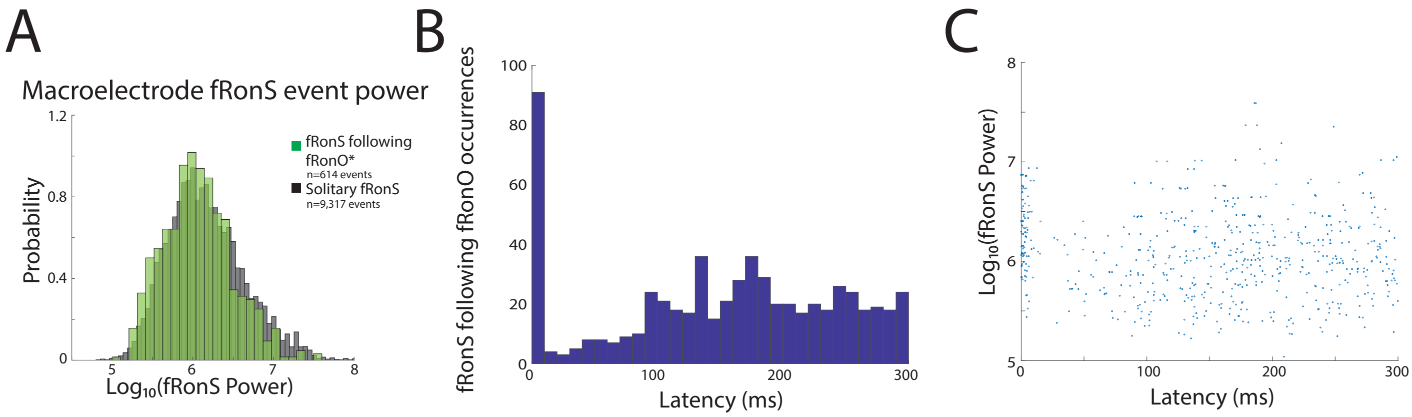

Supplementary Figure 5: An examination of fast ripple on spike (fRonS) following fast ripple on oscillation (fRonO) power with increased temporal resolution. (A) Normalized histogram of fRonS event power in macroelectrode recordings. fRonS power was reduced when it followed a fRonO (t-test, p<1e-5, Cohen's d=0.21) (B) Histogram of the latency in milliseconds (ms) between the fRonO and the after-going fRonS. Note that a distinct subpopulation of fRonS events followed fRonO events by <10 ms. (C) Plot of the fRonS following fRonO power as a function of the latency between the fRonO and the aftergoing fRonS. Note that relatively decreased powers (see A) were seen across a broad range of latencies. However, the power distribution of fRonS events following a fRonO by <10 ms exhibits relatively higher powers as compared to the fRonO power distribution with latencies >10 ms. The difference between these two power distributions suggests that fRonS following fRonO by <10 ms may be generated by a distinct mechanism. Abbreviations: fRonS=fast ripple on spike; ms=millisecond.

Supplementary Table 1: Patient characteristics in the study cohort. Abbreviations M: male, F: female, L: left, R: right, , N/A: not applicable, ATL: anterior temporal lobectomy, MT(L): mesial temporal lobe, MTS: mesial temporal sclerosis, SMA: supplementary motor area, TBI: traumatic brain injury, LOC: loss of consciousness, RNS: responsive neurostimulator, VNS: vagal nerve stimulator, SUDEP: sudden unexpected death in epilepsy, @: time to last follow up.

ID	Risk Factor	MRI	PET (hypo-metabolic)	iEEG clinical consensus SOZs	Surgery	Path.	Outcome
1.	minor TBI	Normal	L temporal	L MT	modified L ATL hippocampus sparing	Gliosis	Engel IA@24 months
2.	hypertensive encephalopathy	post L ATL	N/A	L middle temporal gyrus	modified L temporal lobectomy (posterior temporal included)	Gliosis	Engel 1A @48 months
3.	Minor TBI	Normal	Normal	Right insula, cuneus, inferior and middle frontal gyrus	R. Frontal lobe	Gliosis	Engel IA@24 months
4.	None	Normal	R temporal	R Inferior temporal gyrus	modified R temporal lobectomy (posterior temporal included)	Gliosis	Engel IA@24 months
5.	None	L MTL whitematter hyper-intensity	Normal	R SMA	R frontal lobe resection	cortical dysplasia	Engel IA@24 months
6.	None	Normal	Normal	L cingulate gyrus, medial frontal gyrus, middle frontal gyrus, superior frontal gyrus	L frontal lobectomy	cortical dysplasia	Engel IA@40 months
7.	meningitis	Encephalo-malacia	L temporal	L MT, uncus, superior temporal gyrus, frontal lesion	L temporal and frontal lobe resection	gliosis	Engel IB@42 months
8.	none	1 cm pineal cyst	R lateral temporal	L MT	Modified L temporal lobectomy (posterior temporal included)	Gliosis	Engel IIB@24 months

9.	None	T2 hyper-intensity in R temporal pole > L frontal pole. Inferior portion of R temporal pole with blurred gray-white matter border	R temporal	R MT	R anterior ATL	Cortical dysplasia IIb	Engel IA@60 months
10.	None	Normal	R temporal	Bilateral MT, middle temporal gyrus R>L	modified R ATL (preserved middle and superior temporal gyrus)	gliosis	Engel IVC@48 months
11.	TBI, family history	left superior temporal gyrus encephalomalicia	L parieto-occipital	L temporal neocortical, L frontal	modified LATL hippocampus sparing	gliosis	Engel IV@6 months RNS placed and revised
12.	None	Normal	L temporal	R fusiform gyrus, superior temporal gyrus, uncus	R ATL	MTS	Engel IB@35 months
13.	TBI w/ LOC	L MTS, extra-temporal T2	L temporal and frontal	L MT, fusiform gyrus, uncus	L MT visualase	N/A	Engel IIIA@18 months
14.	None	periventricular nodular heterotopia, right frontal T2	R temporal	R MT	ATL	gliosis	Engel IB@31 months
15.	TBI w/ LOC	Encephalomalacia	R temporal	R insula, bilateral middle temporal gyrus, superior temporal gyrus	modified R ATL (posterior temporal and temporal-parietal-occipital junction included.	gliosis	Engel IVB@33 months

16.	None	Prior R. ATL	N/A	Right orbitofrontal cortex.	R. Frontal lobe	hippocampal sclerosis, cortical dysplasia	Engel IVB@24 months
17.	encephalitis	Encephalomalacia	Normal	L inferior frontal gyrus, insula, MT	L temporal lobe and insula resection	Gliosis	SUDEP @6 weeks
18.	Significant head injury with LOC	Left temporal T2 hyperintensity with mild enhancement	N/A	Bilateral MT, right lateral temporal	L temporal lobectomy, anterior thalamic DBS	Gliosis	Engel IVB@24 months
19.	None	R parietal lobe resection	R parietal and R occipital	R insula, precuneus, middle occipital gyrus, superior parietal lobule, superior occipital gyrus, superior temporal gyrus, middle temporal gyrus	R parietal	gliosis	Engel IIIA@18 months
20.	None	L posterior fossa arachnoid cyst, R ATL	R temporal	L MT, R cingulate, post. cingulate, mesial frontal, precuneus	R anterior cingulate thermal ablation	gliosis	Engel IVB@36 months
21.	None	Prior R parietal resection	R parietal and occipital hypometabolism	R parietal lobe	R. Parietal lobe resection	Gliosis	Engel IVB@36 months
22	TBI	left MTS	normal	L MT	L ATL	gliosis hippocampal sclerosis	Engel IIIA@63 months
23	febrile seizures	prior hippocampal sparing temporal lobectomy	N/A	R anterior cingulate, MT, uncus	R ATL	gliosis	Engel IVB@40 months

Supplementary Table 2: Results of generalized linear mixed effects model for the outcome of the maximum unit firing rate during the fast ripple on oscillation (fRonO) detected in the macroelectrode, (action potentials/sec) after Gaussian smoothing, minus the mean baseline unit firing rate measured 750 msec before the fRonO for each peri-fRonO trial (hfodiff, d.f.=224,266). The random effect terms were patient id, macroelectrode contact ID of the Behnke-Fried electrode, and the unit id to determine that the effects were observed across all units. The fixed effect of the model was whether, in the trial, the fRonO preceded a sharply contoured epileptiform spike, with or without a high-frequency oscilation, within 300 msec ("1" preSpike), or was a solitary fRonO ("0" pre-Spike). Abbreviations CI: confidence interval.

Wilkinson-Roger's Notation: hfodiff ~ 1 + preSPIKE + (1|patient) + (1|electrode) + (1|unit)

fRonO unit firing rate - baseline firing rate Fixed effect name	Estimate [95% CI]	tStat	p-value
(intercept)	0.139 [0.101 0.177]	7.184	<1e-12
1. preSpike	0.044 [0.030 0.58]	6.154	<1e-9

Supplementary Table 3: Results of generalized linear mixed effects model for the outcome of the maximum unit firing rate during the fast ripple on oscillation (fRonO) detected in the macroelectrode, (action potentials/sec) after Gaussian smoothing, minus the mean baseline unit firing rate measured 750 msec before the fRonO for each peri-fRonO trial (hfodiff, d.f.=224,266). The random effect terms were patient id, macroelectrode contact ID of the Behnke-Fried electrode, and the unit id to determine that the effects were observed across all units. The fixed effects of the model were whether, in the trial: 1) the fRonO preceded a sharply contoured epileptiform spike, with or without a high-frequency oscilation, within 300 msec ("1" preSpike) or occurred as a solitary fRonO ("0" preSpike); 2) the unit was sorted as a single unit ("1" sumu) or a multi-unit ("0" sumu). ":" refers to interaction term. Abbreviations CI: confidence interval.

Wilkinson-Roger's Notation: hfodiff ~ 1 + preSPIKE + sumu + sumu:preSPIKE +(1|patient) + (1|electrode) + (1|unit)

Single units trended toward a lower hfodiff with fRonO compared with multi-units. However, preSpike status resulted in a significantly lower hfodiff for single units compared with multi-units.

fRonO unit firing rate - baseline firing rate<br>Fixed effect name	Estimate [95% CI]	tStat	p-value
(intercept)	0.159 [0.114 0.203]	7.00	<1e-11
1. sumu	-0.040 [-0.091 0.010]	-1.5706	0.12
2. preSpike	0.146 [0.122 0.170]	11.84	<1e-31
3. preSpike:sumu	-0.153 [-0.182 -0.123]	-10.147	<1e-23

Supplementary Table 4: Results of generalized linear mixed effects model for the outcome of the maximum unit firing rate during the fast ripple on oscillation (fRonO) detected in the macroelectrode, (action potentials/sec) after Gaussian smoothing, minus the mean baseline unit firing rate measured 750 msec before the fRonO for each peri-fRonO trial (hfodiff, d.f.=224,266). The random effect terms were patient id, macroelectrode contact ID of the Behnke-Fried electrode, and the unit id to determine that the effects were observed across all units. The fixed effects of the model were 1) the log10(power) of the trial's fRonO measured in arbitrary units; and 2) whether, in the trial, the fRonO preceded a sharply contoured epileptiform spike, with or without a high-frequency oscilation, within 300 msec ("1" preSpike), or occurred as a solitary fRonO ("0" preSpike). ":" refers to interaction term. Abbreviations CI: confidence interval.

Wilkinson-Roger's Notation: hfodiff ~ 1 + power + preSPIKE + power:preSPIKE + (1|patient) + (1|electrode) + (1|unit)

The increase in the preSpike fRonO associated peak firing rate, with respect to baseline, (hfodiff) as compared to the hfodiff of solitary fRonO is due to the power:preSpike interaction. Additionally, the pre-spike fRonO had higher power than the solitary fRonO (Figure 2F). Despite this, the preSpike status alone correlated with a decreased hfodiff. preSpike status alone should positively correlate with hfodiff if the solitary fRonO group contained more null trials (hfodiff<=0). These null trials would only influence the power:preSpike interaction if they corresponded to fRonO events with a uniform power distribution. Since artifactual fRonO are more likely to be lower power events, from noise, it is less likely that artifact contamination accounts for the smaller hfodiff in the solitary group.

fRonO unit firing rate - baseline firing rate Fixed effect name	Estimate [95% CI]	tStat	p-value
(intercept)	-0.347 [-0.420 -0.273]	-9.294	<1e-19
1. power	0.0848 [0.752 0.0945]	17.215	<1e-65
2. preSpike	-0.453 [-0.667 -0.238]	-4.133	<1e-4
3. power:preSpike	0.840 [0.048 0.120]	4.508	<1e-5

Supplementary Table 5: Results of generalized linear mixed effects model for the outcome of the mean baseline unit firing rate measured 750 msec before the fast ripple on oscillation (fRonO) for each peri-fRonO trial (bl, d.f.=224,266). The random effect terms were patient id, macroelectrode contact ID of the Behnke-Fried electrode, and the unit id to determine that the effects were observed across all units. The fixed effect of the model was whether, in the trial, the fRonO preceded a sharply contoured epileptiform spike, with or without a high-frequency oscilation, within 300 msec ("1" preSpike) or occurred as a solitary fRonO ("0" preSpike). Abbreviations CI: confidence interval.

Wilkinson-Roger's Notation: bl ~ 1 + preSPIKE + (1|patient) + (1|electrode) + (1|unit)

pre-fRonO<br>baseline unit firing rate	Estimate [95% CI]	tStat	p-value
(intercept)	0.353 [0.244 0.461]	6.350	<1e-9
1. preSpike	0.006 [-0.001 0.012]	1.777	0.076

Supplementary Table 6: Results of generalized linear mixed effects model for the outcome of the maximum unit firing rate during the ripple on oscillation (RonO) detected in the macroelectrode, (action potentials/sec) after Gaussian smoothing, minus the mean baseline unit firing rate measured 750 msec before the RonO for each peri-RonO trial (hfodiff, d.f.=2,603,800). The random effect terms were patient id, macroelectrode contact ID of the Behnke-Fried electrode, and the unit id to determine that the effects were observed across all units. The fixed effect of the model was whether, in the trial, the RonO preceded a sharply contoured epileptiform spike, with or without a high-frequency oscilation, within 300 msec ("1" preSpike), or occurred as a solitary RonO ("0" pre-Spike). Abbreviations CI: confidence interval.

Wilkinson-Roger's Notation: hfodiff ~ 1 + preSPIKE + (1|patient) + (1|electrode) + (1|unit)

RonO unit firing rate - baseline firing rate Fixed effect name	Estimate [95% CI]	tStat	p-value
(intercept)	0.107 [0.077 0.138]	6.97	<1e-11
1. preSpike	0.016 [0.013 0.020]	9.949	<1e-22

Supplementary Table 7: Results of generalized linear mixed effects model for the outcome of the maximum unit firing rate during the ripple on oscillation (RonO) detected in the macroelectrode, (action potentials/sec) after Gaussian smoothing, minus the mean baseline unit firing rate measured 750 msec before the RonO for each peri-RonO trial (hfodiff, d.f.=2,603,845). The random effect terms were patient id, macroelectrode contact ID of the Behnke-Fried electrode, and the unit id to determine that the effects were observed across all units. The fixed effects of the model were 1) the log10(power) of the trial's RonO measured in arbitrary units, and 2) whether, in the trial, the RonO preceded a sharply contoured epileptiform spike, with or without a high-frequency oscilation, within 300 msec (preSpike "1") or occurred as a solitary RonO (preSpike "0") . ":" refers to interaction term. Abbreviations CI: confidence interval.

Wilkinson-Roger's Notation: hfodiff ~ 1 + power + preSPIKE + power:preSPIKE + (1|patient) + (1|electrode) + (1|unit)

The increase in the preSpike RonO associated peak firing rate, with respect to baseline, (hfodiff) as compared to the hfodiff of solitary RonO is due to the power:preSpike interaction. Additionally, the pre-spike RonO had higher power than the solitary RonO (Figure 3D). Despite this, the preSpike status alone correlated with a decreased hfodiff.

RonO unit firing rate - baseline firing rate<br>Fixed effect name	Estimate [95% CI]	tStat	p-value
(intercept)	-0.378 [-0.421 -0.336]	-17.304	<1e-66
1. power	0.075 [0.072 0.078]	50.398	<1e-99
2. preSpike	-0.095 [-0.167 -0.025]	-2.637	<0.01
3. preSpike:power	0.016 [0.006 0.026]	2.998	<0.01

Supplementary Table 8: Results of generalized linear mixed effects model for the outcome of the mean baseline unit firing rate measured 750 msec before the ripple on oscillation (RonO) for each peri-RonO trial (bl, d.f.=2,603,845). The random effect terms were patient id, macroelectrode contact ID of the Behnke-Fried electrode, and the unit id to determine that the effects were observed across all units. The fixed effect of the model was whether, in the trial, the RonO preceded a sharply contoured epileptiform spike, with or without a high-frequency oscilation, within 300 msec (preSpike "1") or occurred as a solitary RonO (preSpike "0"). Abbreviations CI: confidence interval.

Wilkinson-Roger's Notation: bl ~ 1 + preSPIKE + (1|patient) + (1|electrode) + (1|unit)

pre-fRonO<br>baseline unit firing rate	Estimate [95% CI]	tStat	p-value
(intercept)	0.270 [0.191 0.350]	6.67	<1e-10
1. preSpike	0.008 [0.006 0.009]	9.979	<1e-22

Supplementary Table 9: Results of generalized linear mixed effects model for the outcome of the mean baseline unit firing rate measured 750 msec before the ripple on oscillation (RonO) for each peri-RonO trial (bl, d.f.=2,603,845). The random effect terms were patient id, macroelectrode contact ID of the Behnke-Fried electrode, and the unit id to determine that the effects were observed across all units. The fixed effects of the model were 1) the log10(power) of the trial's RonO measured in arbitrary units, and 2) whether, in the trial, the RonO preceded a sharply contoured epileptiform spike, with or without a high-frequency oscilation, within 300 msec (preSpike "1") or occurred as a solitary RonO (preSpike "0"). ":" refers to interaction term. Abbreviations CI: confidence interval.

Wilkinson-Roger's Notation: bl ~ 1 + power + preSPIKE + soz + power:preSPIKE + soz:power + soz:power:preSPIKE + soz:preSPIKE + (1|patient) + (1|electrode) + (1|unit)

The increase in baseline firing rate in the preSpike group is no longer evident after including power as a fixed effect.

pre-RonO<br>baseline unit firing rate	Estimate [95% CI]	tStat	p-value
(intercept)	0.241 [0.160 0.321]	5.872	<1e-8
1. power	0.005 [0.003 0.006]	6.281	<1e-10
2. preSpike	0.030 [-0.005 0.064]	1.689	0.09
3. preSpike:power	-0.003 [-0.008 0.002]	-1.249	0.212

Supplementary Table 10: Results of generalized linear mixed effects model for the outcome of the maximum unit firing rate during the fast ripple on spike (fRonS) detected in the macroelectrode, (action potentials/sec) after Gaussian smoothing, minus the mean baseline unit firing rate measured 750 msec before the fRonS for each peri-fRonS trial (hfodiff, d.f.=48,164). The random effect terms were patient id, macroelectrode contact ID of the Behnke-Fried electrode, and the unit id to determine that the effects were observed across all units. The fixed effect of the model were 1) whether, in the trial, the fRonS followed a fast ripple on oscillation (fRonO) within 300 msec (followingfRonO "1") or occurred not following a fRonO (followingfRonO "0"); and 2) whether, in the trial, the fRonS followed a ripple on oscillation (RonO) within 300 msec (followingRonO "1") or occurred not following a RonO (followingRonO "0"). ":" refers to interaction term. Abbreviations CI: confidence interval.

Wilkinson-Roger's Notation: hfodiff ~ 1 + followingfRonO + followingRonO + followingfRonO:followingRonO + (1|patient) + (1|electrode) + (1|unit)

fRonS unit firing rate - baseline firing rate Fixed effect name	Estimate [95% CI]	tStat	p-value
(intercept)	0.587 [0.312 0.861]	4.191	<1e-4
2. followingRonO	0.056 [0.032 0.081]	4.550	<1e-5
3. followingfRonO	-0.203 [-0.302 -0.103]	-4.009	<1e-4
6. followingRonO:following-fRonO	0.098 [-0.013 0.209]	1.729	0.08

Supplementary Table 11: Results of generalized linear mixed effects model for the outcome of the maximum unit firing rate during the fast ripple on spike (fRonS) detected in the macroelectrode, (action potentials/sec) after Gaussian smoothing, minus the mean baseline unit firing rate measured 750 msec before the fRonS for each peri-fRonS trial (hfodiff, d.f.=48,164). The random effect terms were patient id, macroelectrode contact ID of the Behnke-Fried electrode, and the unit id to determine that the effects were observed across all units. The fixed effects of the model were 1) the log10(power) of the trial's fRonS measured in arbitrary units; 2) whether, in the trial, the fRonS followed a fast ripple on oscillation (fRonO) within 300 msec (followingfRonO "1") or occurred not following a fRonO (followingfRonO "0"); and 3) whether, in the trial, the fRonS followed a ripple on oscillation (RonO) within 300 msec (followingRonO "1") or occurred not following a RonO (followingRonO "0"). ":" refers to interaction term. Abbreviations CI: confidence interval.

Wilkinson-Roger's Notation: hfodiff ~ 1 + power + followingfRonO + followingRonO + power:followingfRonO + power:followingRonO + followingfRonO:followingRonO + power:followingfRonO:followingRonO + (1|patient) + (1|electrode) + (1|unit)

Decreased peak firing with respect to baseline (hfodiff) in fRonS followingfRonO, relative to solitary fRonS, is due to followingfRonO status correlating with decrease hfodiff, and a decrease in following fRonO fRonS power with respect to mean solitary fRonS power (Figure 4C). Increased hfodiff in fRonS followingRonO, relative to solitary fRonS, is due to the followingRonO status positively correlated with hfodiff.

fRonS unit firing rate - baseline firing rate<br>Fixed effect name	Estimate [95% CI]	tStat	p-value
(intercept)	-0.668 [-0.970 -0.366]	-4.332	<1e-4
1. power	0.201 [0.179 0.223]	17.808	<1e-69
2. followingRonO	0.956 [0.683 1.227]	6.888	<1e-11
3. followingfRonO	-1.561 [-2.944 -0.180]	-2.215	0.02
4. power:followingRonO	-0.144 [-0.187 -0.101]	-6.546	<1e-10
5. power:followingfRonO	0.230 [0.002 0.459]	1.975	0.05
6. followingRonO:followingfRonO	0.382 [-1.142 1.905]	0.490	0.62
7. power:followingRonO:followingfRonO	-0.056 [-0.310 0.195]	-0.435	0.66

Supplementary Table 12: Results of generalized linear mixed effects model for the outcome of the mean baseline unit firing rate measured 750 msec before the fast ripple on spike (fRonS) for each peri-fRonS trial (bl, d.f.=48,164). The random effect terms were patient id, macroelectrode contact ID of the Behnke-Fried electrode, and the unit id to determine that the effects were observed across all units. The fixed effects of the model were 1) whether, in the trial, the fRonS followed a fast ripple on oscillation (fRonO) within 300 msec (followingfRonO "1") or occurred not following a fRonO (followingfRonO "0"); and 2) whether, in the trial, the fRonS followed a ripple on oscillation (RonO) within 300 msec (followingRonO "1") or occurred not following a RonO (followingRonO "0"). ":" refers to interaction term. Abbreviations CI: confidence interval.

Wilkinson-Roger's Notation: bl ~ 1 + followingfRonO + followingRonO + followingfRonO:followingRonO + (1|patient) + (1|electrode) + (1|unit)

fRonS unit firing rate - baseline firing rate Fixed effect name	Estimate [95% CI]	tStat	p-value
(intercept)	0.302 [0.175 0.428]	4.681	<1e-5
1. followingRonO	0.015 [0.008 0.022]	4.209	<1e-4
2. followingfRonO	-0.004 [-0.032 0.241]	-0.281	.78
3. followingRonO:following-fRonO	-0.020 [-0.050 0.011]	-1.22	0.22

Supplementary Table 13: Results of generalized linear mixed effects model for the outcome of the mean baseline unit firing rate measured 750 msec before the fast ripple on spike (fRonS) for each peri-fRonS trial (bl, d.f.=48,164). The random effect terms were patient id, macroelectrode contact ID of the Behnke-Fried electrode, and the unit id to determine that the effects were observed across all units. The fixed effects of the model were 1) the log10(power) of the trial's fRonS measured in arbitrary units; 2) whether, in the trial, the fRonS followed a fast ripple on oscillation (fRonO) within 300 msec (followingfRonO "1") or occurred not following a fRonO (followingfRonO "0"; and 3) whether, in the trial, the fRonS followed a ripple on oscillation (RonO) within 300 msec (followingRonO "1") or occurred not following a RonO (followingRonO "0"). ":" refers to interaction term. Abbreviations CI: confidence interval.

Wilkinson-Roger's Notation: bl ~ 1 + power + followingfRonO + followingRonO + power:followingfRonO + power:followingRonO + followingfRonO:followingRonO + power:followingfRonO:followingRonO + (1|patient) + (1|electrode) + (1|unit)

Increased baseline firing prior to fRonS following RonO is no longer evident after including power as a fixed effect.

fRonS unit firing rate - baseline firing rate<br>Fixed effect name	Estimate [95% CI]	tStat	p-value
(intercept)	0.327 [0.195 0.460]	4.843	<1e-5
1. power	-0.004 [-0.010 0.002]	-1.284	0.2
2. followingRonO	-0.040 [-0.117 0.038]	-1.001	0.32
3. followingfRonO	-0.040 [-0.117 0.038]	-1.291	0.197
4. power:followingRonO	-0.260 [-0.004 0.021]	1.383	0.166
5. power:followingfRonO	0.042 [-0.023 0.107]	1.271	0.20
6. followingRonO:following-fRonO	0.177 [-0.257 0.611]	0.800	0.42
7. power:followingRonO:fol-lowingfRonO	-0.033 [-0.104 0.039]	-0.895	0.37

Supplementary Table 14: Results of generalized linear mixed effects model for the outcome of the maximum unit firing rate during the ripple on spike (RonS) detected in the macroelectrode, (action potentials/sec) after Gaussian smoothing, minus the mean baseline unit firing rate measured 750 msec before the RonS for each peri-RonS trial (hfodiff, d.f.=391,660). The random effect terms were patient id, macroelectrode contact ID of the Behnke-Fried electrode, and the unit id to determine that the effects were observed across all units. The fixed effect of the model were 1) whether, in the trial, the RonS followed a fast ripple on oscillation (fRonO) within 300 msec (followingfRonO "1") or occurred not following a fRonO (followingfRonO "0"); and 3) whether, in the trial, the fRonS followed a ripple on oscillation (RonO) within 300 msec (followingRonO "1") or occurred not following a RonO (followingRonO "0"). ":" refers to interaction term. Abbreviations CI: confidence interval.

Wilkinson-Roger's Notation: hfodiff ~ 1 + followingfRonO + followingRonO + followingfRonO:followingRonO

fRonS unit firing rate - baseline firing rate<br>Fixed effect name	Estimate [95% CI]	tStat	p-value
(intercept)	0.392 [0.263 0.521]	5.951	<1e-8
2. followingRonO	0.003 [-0.004 0.010]	0.716	0.47
3. followingfRonO	-0.033 [-0.062 -0.003]	-2.192	0.03
6. followingRonO:following-fRonO	0.023 [-0.011 0.057]	1.331	0.18

Supplementary Table 15: Results of generalized linear mixed effects model for the outcome of the maximum unit firing rate during the ripple on spike (RonS) detected in the macroelectrode, (action potentials/sec) after Gaussian smoothing, minus the mean baseline unit firing rate measured 750 msec before the RonS for each peri-RonS trial (hfodiff, d.f.=391,660). The random effect terms were patient id, macroelectrode contact ID of the Behnke-Fried electrode, and the unit id to determine that the effects were observed across all units. The fixed effects of the model were 1) the log10(power) of the trial's RonS measured in arbitrary units; 2) whether, in the trial, the RonS followed a fast ripple on oscillation (fRonO) within 300 msec (followingfRonO "1") or occurred not following a fRonO (followingfRonO "0"); and 3) whether, in the trial, the fRonS followed a ripple on oscillation (RonO) within 300 msec (followingRonO "1") or occurred not following a RonO (followingRonO "0"). ":" refers to interaction term. Abbreviations CI: confidence interval.

Wilkinson-Roger's Notation: hfodiff ~ 1 + power + followingfRonO + followingRonO + power:followingfRonO + power:followingRonO + followingfRonO:followingRonO + power:followingfRonO:followingRonO + (1|patient) + (1|electrode) + (1|unit)

Decreased peak firing with respect to baseline (hfodiff) in followingfRonO RonS, relative to solitary RonS, is due to the interaction of followingfRonO status and power negatively correlating with hfodiff.

fRonS unit firing rate - baseline firing rate<br>Fixed effect name	Estimate [95% CI]	tStat	p-value
(intercept)	-0.387 [-0.529 -0.245]	-5.356	<1e-7
1. power	0.113 [0.107 0.120]	33.982	<1e-100
2. followingRonO	-0.149 [-0.248 -0.049]	-2.93	<0.01
3. followingfRonO	0.472 [.146 0.799]	2.833	<0.01
4. power:followingRonO	0.023 [0.009 0.037]	3.131	<0.01
5. power:followingfRonO	-0.072 [-0.116 -0.027]	-3.140	<0.01
6. followingRonO:followingfRonO	-0.360 [-0.757 0.038]	-1.773	0.07
7. power:followingRonO:followingfRonO	0.055 [-0.0001 0.11]	1.956	0.05

Supplementary Table 16: Results of generalized linear mixed effects model for the outcome of the mean baseline unit firing rate measured 750 msec before the ripple on spike (RonS) for each peri-RonS trial (bl, d.f.=391,660). The random effect terms were patient id, macroelectrode contact ID of the Behnke-Fried electrode, and the unit id to determine that the effects were observed across all units. The fixed effects of the model were 1) whether, in the trial, the RonS followed a fast ripple on oscillation (fRonO) within 300 msec (followingfRonO "1") or occurred not following a fRonO (followingfRonO "0"); and 2) whether, in the trial, the fRonS followed a ripple on oscillation (RonO) within 300 msec (followingRonO "1") or occurred not following a RonO (followingRonO "0"). ":" refers to interaction term. Abbreviations CI: confidence interval.

Wilkinson-Roger's Notation: bl ~ 1 + followingfRonO + followingRonO + followingfRonO:followingRonO + (1|patient) + (1|electrode) + (1|unit)

fRonS unit firing rate - baseline firing rate Fixed effect name	Estimate [95% CI]	tStat	p-value
(intercept)	0.319 [0.193 0.445]	4.932	<1e-6
1. followingRonO	0.016 [0.013 0.018]	13.409	<1e-40
2. followingfRonO	0.009 [-0.0002 0.019]	1.902	0.06
3. followingRonO:following-fRonO	-0.018 [-0.03 -0.008]	-3.317	<1e-3

Supplementary Table 17: Results of generalized linear mixed effects model for the outcome of the mean baseline unit firing rate measured 750 msec before the fast ripple on spike (RonS) for each peri-RonS trial (bl, d.f.=391,660). The random effect terms were patient id, macroelectrode contact ID of the Behnke-Fried electrode, and the unit id to determine that the effects were observed across all units. The fixed effects of the model were 1) the log10(power) of the trial's RonS measured in arbitrary units; 2) whether, in the trial, the RonS followed a fast ripple on oscillation (fRonO) within 300 msec (followingfRonO "1") or occurred not following a fRonO (followingfRonO "0"); and 3) whether, in the trial, the RonS followed a ripple on oscillation (RonO) within 300 msec (followingRonO "1") or occurred not following a RonO (followingRonO "0"). ":" refers to interaction term. Abbreviations CI: confidence interval.

Wilkinson-Roger's Notation: bl ~ 1 + power + followingfRonO + followingRonO + power:followingfRonO + power:followingRonO + followingfRonO:followingRonO + power:followingfRonO:followingRonO + (1|patient) + (1|electrode) + (1|unit)

After adding power as a fixed effect the increase in baseline firing rate for RonS followingRonO remains and is

fRonS unit firing rate - baseline firing rate Fixed effect name	Estimate [95% CI]	tStat	p-value
(intercept)	0.337 [0.209 0.464]	5.178	<1e-6
1. power	-0.003 [-0.005 -0.0004]	-2.376	0.02
2. followingRonO	0.034 [0.002 0.066]	2.060	0.04
3. followingfRonO	-0.152 [-0.258 -0.047]	-2.826	0.005
4. power:followingRonO	-0.003 [-0.007 0.002]	-1.132	0.25
5. power:followingfRonO	0.022 [0.008 0.037]	3.014	<0.01
6. followingRonO:following-fRonO	0.001 [-0.127 0.130]	0.020	0.98
7. power:followingRonO:fol-lowingfRonO	-0.002 [-0.020 0.016]	-0.205	0.84
